# Rising CO_2_ Levels Will Intensify Phytoplankton Blooms in Eutrophic and Hypertrophic Lakes

**DOI:** 10.1371/journal.pone.0104325

**Published:** 2014-08-13

**Authors:** Jolanda M. H. Verspagen, Dedmer B. Van de Waal, Jan F. Finke, Petra M. Visser, Ellen Van Donk, Jef Huisman

**Affiliations:** 1 Department of Aquatic Microbiology, Institute for Biodiversity and Ecosystem Dynamics, University of Amsterdam, Amsterdam, The Netherlands; 2 Department of Aquatic Ecology, Netherlands Institute of Ecology, Wageningen, The Netherlands; 3 Institute of Environmental Biology, University of Utrecht, Utrecht, The Netherlands; University of Connecticut, United States of America

## Abstract

Harmful algal blooms threaten the water quality of many eutrophic and hypertrophic lakes and cause severe ecological and economic damage worldwide. Dense blooms often deplete the dissolved CO_2_ concentration and raise pH. Yet, quantitative prediction of the feedbacks between phytoplankton growth, CO_2_ drawdown and the inorganic carbon chemistry of aquatic ecosystems has received surprisingly little attention. Here, we develop a mathematical model to predict dynamic changes in dissolved inorganic carbon (DIC), pH and alkalinity during phytoplankton bloom development. We tested the model in chemostat experiments with the freshwater cyanobacterium *Microcystis aeruginosa* at different CO_2_ levels. The experiments showed that dense blooms sequestered large amounts of atmospheric CO_2_, not only by their own biomass production but also by inducing a high pH and alkalinity that enhanced the capacity for DIC storage in the system. We used the model to explore how phytoplankton blooms of eutrophic waters will respond to rising CO_2_ levels. The model predicts that (1) dense phytoplankton blooms in low- and moderately alkaline waters can deplete the dissolved CO_2_ concentration to limiting levels and raise the pH over a relatively wide range of atmospheric CO_2_ conditions, (2) rising atmospheric CO_2_ levels will enhance phytoplankton blooms in low- and moderately alkaline waters with high nutrient loads, and (3) above some threshold, rising atmospheric CO_2_ will alleviate phytoplankton blooms from carbon limitation, resulting in less intense CO_2_ depletion and a lesser increase in pH. Sensitivity analysis indicated that the model predictions were qualitatively robust. Quantitatively, the predictions were sensitive to variation in lake depth, DIC input and CO_2_ gas transfer across the air-water interface, but relatively robust to variation in the carbon uptake mechanisms of phytoplankton. In total, these findings warn that rising CO_2_ levels may result in a marked intensification of phytoplankton blooms in eutrophic and hypertrophic waters.

## Introduction

Since the start of the industrial revolution, atmospheric CO_2_ concentrations have increased from 275 to 400 ppm CO_2_, and climate change scenarios predict that atmospheric CO_2_ will further increase [1]. Enhanced dissolution of CO_2_ will lower the pH of aquatic ecosystems [2], [3]. However, CO_2_ in freshwater ecosystems does not only originate from dissolution of atmospheric CO_2_, but also from mineralization of organic carbon obtained from terrestrial sources in the surrounding watershed [4]. Mineralization of organic carbon causes CO_2_ supersaturation in many lakes, in some cases even reaching CO_2_ levels exceeding 10,000 ppm [5]–[7].

Phytoplankton fix CO_2_ for photosynthesis, and many species can also utilize bicarbonate as a carbon source [8]–[10]. Assimilation of inorganic carbon by dense phytoplankton blooms can deplete the dissolved CO_2_ concentration [11]–[15], sometimes down to levels below 1 ppm [7], [15], so that these waters become severely CO_2_-undersaturated. CO_2_ depletion will cause an increase in pH [11], [16], [17]. Indeed, in eutrophic lakes with dense phytoplankton blooms, pH easily exceeds values of 9 [7], [15], and can reach values as high as 11 in shallow hypertrophic lakes [18].

The combination of high pH values and CO_2_ depletion in freshwaters is often associated with cyanobacterial blooms [19], [20]. Several of the cyanobacterial species that commonly dominate these blooms are capable of producing toxic substances [21], [22]. Consequently, cyanobacterial blooms threaten the water quality of many freshwater lakes and brackish waters around the world, including Lake Erie in USA-Canada [23], Lake Taihu in China [24], [25], Lake Biwa in Japan [26], Lake Victoria in Africa [27], [28], the Baltic Sea in Northern Europe [29], [30], and many other ecologically and economically important lakes, rivers and estuaries [21], [22], [31], [32]. Cyanobacterial blooms are expected to benefit from global warming [32]–[35]. The response of cyanobacteria to rising CO_2_ concentrations, however, is less well understood, although it is clear that there is a strong interaction between cyanobacterial bloom development and CO_2_ availability.

**Figure 1:**
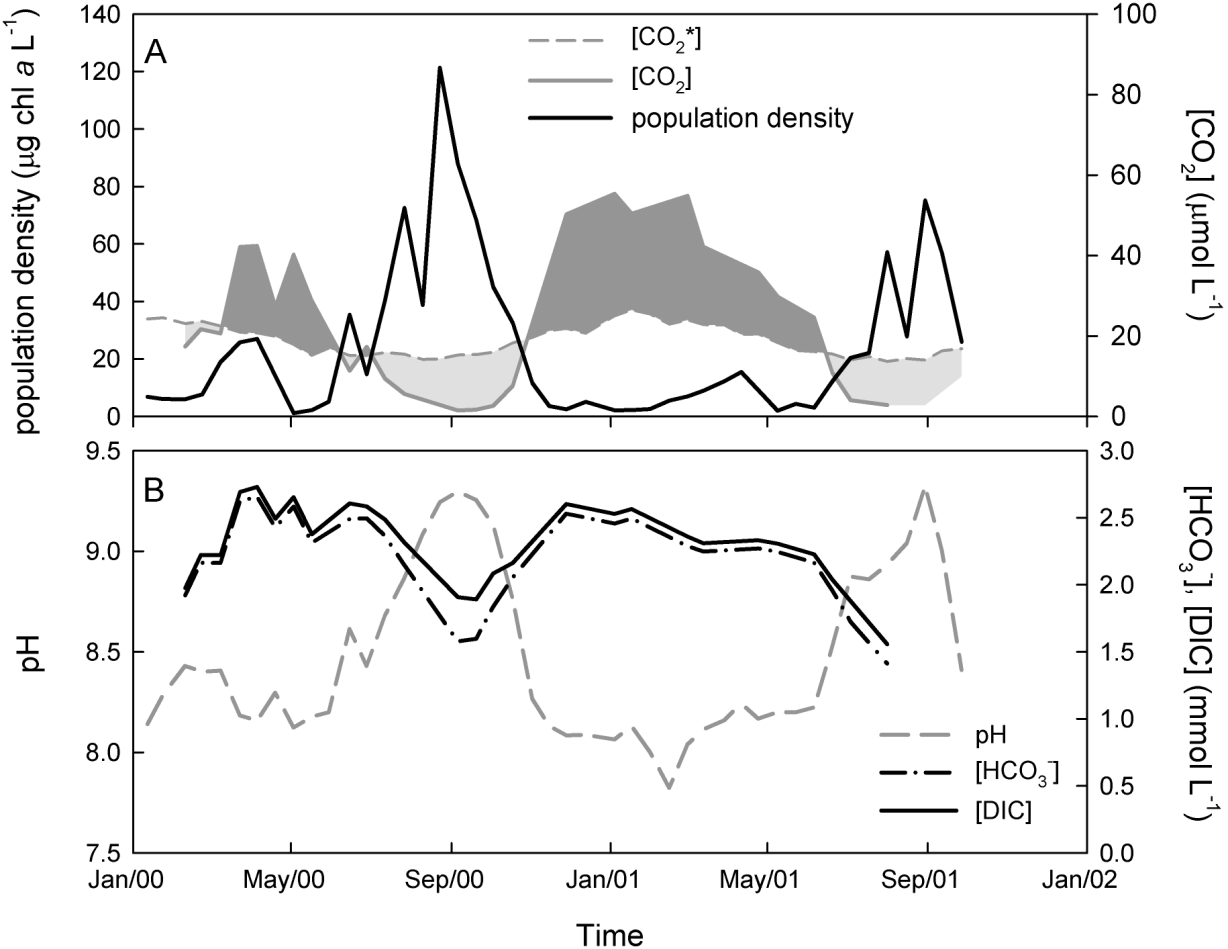
Seasonal dynamics of phytoplankton blooms in Lake Volkerak. (A) Changes in phytoplankton population density (strongly dominated by the cyanobacterium *Microcystis*) and measured dissolved CO_2_ concentration ([CO_2_]) during two consecutive years. The dashed line is the expected dissolved CO_2_ concentration ([CO_2_*]) when assuming equilibrium with atmospheric pCO_2_. Dark shading indicates that the lake is supersaturated with CO_2_, while light shading indicates undersaturation. (B) Changes in pH, bicarbonate and total DIC concentration. Sampling details are described in Text S1.

As an illustration, Fig. 1 provides data from Lake Volkerak, a large eutrophic lake in The Netherlands [31], [36]. In winter and spring, CO_2_ concentrations in Lake Volkerak largely exceed the CO_2_ concentrations that would be predicted from equilibrium with the atmosphere, and hence the lake is supersaturated with CO_2_. In summer and early fall, however, Lake Volkerak is covered by dense blooms of the harmful cyanobacterium *Microcystis* [31], [36]. The photosynthetic activity of these blooms depletes the CO_2_ concentration, such that the lake becomes undersaturated with CO_2_ in summer while the pH rises to values above 9 for several months (Fig. 1, Text S1).

Hence, there is a strong and complex coupling between phytoplankton growth and the inorganic carbon chemistry of aquatic ecosystems that may lead to CO_2_ depletion during dense blooms, even in lakes that would otherwise be supersaturated with CO_2_. This biological-chemical coupling is further complicated by several additional feedbacks. For instance, dense phytoplankton blooms not only deplete CO_2_ and enhance pH but also increase the turbidity of the water column as a result of self-shading, thereby reducing light available for carbon fixation by photosynthesis [31], [37]. Moreover, nutrient uptake by dense blooms also affects alkalinity [38]–[40], which in turn feeds back upon pH and the speciation of dissolved inorganic carbon (DIC). Given the pH and total DIC concentration, it is straightforward to calculate the CO_2_, bicarbonate and carbonate concentrations [41]–[43]. However, we still lack an integrative understanding that incorporates the different feedback loops to enable quantitative prediction of the changes in DIC concentration and pH during phytoplankton bloom development. Yet, such an integrative approach will be required to assess how rising CO_2_ concentrations will affect phytoplankton blooms and carbon sequestration in aquatic systems.

In this study, we investigate the dynamic feedbacks between phytoplankton growth, DIC, alkalinity, pH and light during phytoplankton bloom development. Our study specifically focuses on eutrophic and hypertrophic waters, where an excess of mineral nutrients provides ideal conditions for phytoplankton blooms. We incorporate standard inorganic carbon chemistry into a mathematical model of phytoplankton growth with CO_2_, bicarbonate and light as limiting resources. We test the model in controlled laboratory experiments at different pCO_2_ levels and alkalinities using the harmful cyanobacterium *Microcystis aeruginosa*, a cosmopolitan and often toxic species that develops dense blooms in Lake Volkerak and many other eutrophic lakes worldwide [23]–[27], [31], [33]. Our model fits were in good agreement with the experimental results, and show that the coupling between phytoplankton growth and inorganic carbon chemistry is strongly affected by the CO_2_ level. Subsequently, we use the experimentally validated model to explore how phytoplankton blooms in eutrophic lakes may respond to rising CO_2_ availability.

## The Model

### General outline

Our model builds upon a long tradition of model studies in phytoplankton ecology [44]–[49], extending these earlier studies by the incorporation of dynamic changes in inorganic carbon availability, alkalinity and pH induced by phytoplankton blooms. The model considers a well-mixed water column, illuminated from above, with a growing phytoplankton population that is homogeneously distributed over depth. Here we introduce the key assumptions, while the model is described in full detail in Text S2 (for chemostats) and Text S3 (for lakes).

### Phytoplankton population dynamics

In this study, we focus on eutrophic and hypertrophic ecosystems where all nutrients are in excess. Hence, the specific growth rate of phytoplankton does not become limited by nutrients but depends only on its cellular carbon content. The cellular carbon content is a dynamic variable, which increases by the photosynthetically-driven uptake of CO_2_ and bicarbonate, while it decreases by respiration and by dilution of the cellular carbon content due to population growth. More precisely, let *X* denote the population density of the phytoplankton, and let *Q* denote its cellular carbon content. Changes in phytoplankton population density and its carbon content can then be described by:
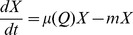
(1)

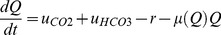
(2)where *µ*(*Q*) is the specific growth rate of the phytoplankton as function of its cellular carbon content, *m* is the specific loss rate (e.g., by background mortality, grazing, sedimentation), *u_CO2_* and *u_HCO3_* are the uptake rates of CO_2_ and bicarbonate, respectively, and *r* is the respiration rate.

We assume that the specific growth rate increases with the cellular carbon content of the phytoplankton, which require a minimum cellular carbon content in order to function (i.e., *µ*(*Q_MIN_*) = 0) and reach their maximum specific growth rate when satiated with carbon (i.e., *µ*(*Q_MAX_*) = *µ_MAX_*). Uptake rates of CO_2_ (*u_CO2_*) and bicarbonate (*u_HCO3_*) are increasing but saturating functions of the ambient CO_2_ and bicarbonate concentration according to Michaelis-Menten kinetics, and are suppressed when cells become satiated with carbon [50]. The energy for carbon assimilation comes from photosynthesis, and therefore depends on light availability. The underwater light environment is described by Lambert-Beer’s law, taking into account that a growing phytoplankton population gradually increases the turbidity of the water column through self-shading and thereby reduces the light available for further photosynthesis [31], [51]. We assume that the respiration rate (*r*) increases with the cellular carbon content, approaching maximum values when cells become satiated with carbon [52]. The mathematical equations describing these relationships are presented in Text S2.

To assess to what extent phytoplankton growth is limited by carbon, we introduce a simple relative measure of the inorganic carbon availability for photosynthesis (*f_C_*):
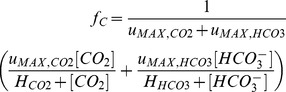
(3)where *u_MAX,CO2_* and *u_MAX,HCO3_* are the maximum uptake rates of carbon dioxide and bicarbonate, respectively, and *H_CO2_* and *H_HCO3_* are their half-saturation constants. We note that 0≤*f_C_*≤1. The level of carbon limitation (*L_C_*) can then be defined as the reduction in carbon uptake due to low carbon availability: *L_C_* = (1−*f_C_*)×100%. Accordingly, if CO_2_ and bicarbonate are both available in saturating concentrations, *L_C_* will be close to 0%. Conversely, if CO_2_ and bicarbonate are available only in trace amounts, *L_C_* approaches 100%.

### Dissolved inorganic carbon, alkalinity and pH

On the timescales used in our model (ranging from minutes to days) the speciation of dissolved inorganic carbon is essentially in equilibrium with alkalinity and pH. Therefore, let [DIC] denote the total concentration of dissolved inorganic carbon. Changes in [DIC] can be described by:
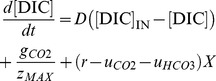
(4)


The first term on the right-hand side of Eqn (4) describes changes in the DIC concentration due to the influx ([DIC]_IN_) and efflux of water containing DIC, where *D* is the dilution rate. The second term describes exchange of CO_2_ gas with the atmosphere, where *g_CO2_* is the CO_2_ flux across the air-water interface (also known as the carbon sequestration rate) and division by *z_MAX_* converts the CO_2_ flux per unit surface into a volumetric CO_2_ change. The third term describes how the DIC concentration increases through respiration (*r*) and decreases through uptake of CO_2_ (*u_CO2_*) and bicarbonate (*u_HCO3_*) by phytoplankton.

The CO_2_ flux across the air-water interface is proportional to the difference between the dissolved CO_2_ concentration that would be attained in equilibrium with the atmospheric pressure ([CO_2_*]) and the actual dissolved CO_2_ concentration [53], [54]:
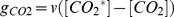
(5)where *v* is the gas transfer velocity. The equilibrium value [CO_2_*] is calculated from Henry’s law, i.e., [CO_2_*] = K_0_ pCO_2_, where pCO_2_ is the partial pressure of CO_2_ in air and K_0_ is the solubility constant of CO_2_ gas in water. In our experiments, gas exchange will increase with the gas flow rate (*a*). Hence, we assume *v* = *b a*, where *b* is a constant of proportionality reflecting the efficiency of gas exchange.

Changes in pH depend on alkalinity, which is a measure of the acid-neutralizing capacity of water. In our experiments, alkalinity is dominated by dissolved inorganic carbon and inorganic phosphates [40]:
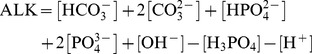
(6)


We note from Eqn (6) that changes in the concentration of dissolved CO_2_ do not change alkalinity. Furthermore, uptake of bicarbonate for photosynthesis is accompanied by the release of a hydroxide ion or uptake of a proton, and therefore does not change alkalinity either. Hence, carbon assimilation by phytoplankton does not affect alkalinity [40]. However, nitrate, phosphate and sulfate assimilation are accompanied by proton consumption to maintain charge balance, and thus increase alkalinity [38]–[40]. More specifically, both nitrate and phosphate uptake increase alkalinity by 1 mole equivalent, whereas sulfate uptake increases alkalinity by 2 mole equivalents [40]. Hence, changes in alkalinity can be described as:
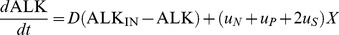
(7)where ALK_IN_ is the alkalinity of the water influx, and *u_N_*, *u_P_* and *u_S_* are the uptake rates of nitrate, phosphate and sulfate by the growing phytoplankton population. The model keeps track of the nitrate, phosphate and sulfate concentration.

At each time step, the dissolved CO_2_, bicarbonate and carbonate concentration and pH are calculated from [DIC] and alkalinity (Text S2).

## Materials and Methods

### Experiments

#### Experimental system

We tested the model using two strains of the freshwater cyanobacterium *Microcystis aeruginosa*. Strain *Microcystis* CYA140 was obtained from the Norwegian Institute for Water Research (NIVA). Strain *Microcystis* HUB5-2-4 was obtained from the Humboldt University of Berlin, Germany. Both *Microcystis* strains grow as single cell populations. Although all culture equipment was autoclaved prior to the experiments, we were not able to sustain axenic conditions. However, regular microscopic inspection confirmed that abundances of heterotrophic bacteria remained low (<0.1% of the total biomass) for the entire duration of the experiments.

The experiments were carried out in laboratory-built chemostats specifically designed for phytoplankton studies [49], [55], [56]. Each chemostat consisted of a flat culture vessel illuminated from one side with a constant incident light intensity of *I_IN_* = 50±1 µmol photons m^−2 ^s^−1^ provided by white fluorescent tubes (Philips PL-L 24W/840/4P, Philips Lighting, Eindhoven, The Netherlands). The chemostats had an optical path length (“mixing depth”) of *z_MAX_* = 5 cm, and an effective working volume of 1.7 L. The chemostats were supplied with a nutrient-rich mineral medium [57] to prevent nutrient limitation during the experiments. Under conditions of nutrient excess, phytoplankton population densities tend to become much higher in laboratory chemostats where phytoplankton is concentrated within only 5 cm depth than in lakes where the phytoplankton population is dispersed over several meters depth [51], [58]. This scaling rule implies that nutrient concentrations have to be much higher in mineral media of small-scale laboratory chemostats than in eutrophic lakes to sustain these high population densities. The chemostats were maintained at a constant temperature using a metal cooling finger connected to a Colora thermocryostat, and were aerated with sterilized (0.2 µm Millex-FG Vent Filter, Millipore, Billerica, MA, USA) N_2_ gas enriched with different CO_2_ concentrations using Brooks Mass Flow Controllers (Brooks Instrument, Hatfield, PA, USA). The gas mixture was dispersed from the bottom of the chemostat vessel in fine bubbles at a constant gas flow rate (*a*) of 25 L h^−1^.

#### Treatments

First, we studied dynamic changes in inorganic carbon chemistry and pH in six chemostats without any phytoplankton, to assess whether the model adequately described the dissolution of CO_2_ and subsequent dynamic changes in inorganic carbon chemistry. These auxiliary experiments are described in Text S4.

Subsequently, we ran two chemostat experiments with *Microcystis* CYA140 to investigate dynamic changes in phytoplankton growth, inorganic carbon chemistry, alkalinity and pH. The first chemostat was provided with a low pCO_2_ of 200 ppm in the gas flow and 0.5 mmol L^−1^ NaHCO_3_ in the mineral medium. The second chemostat was provided with a high pCO_2_ of 1,200 ppm in the gas flow and 2.0 mmol L^−1^ NaHCO_3_ in the mineral medium. Both chemostats had a dilution rate of *D* = 0.011 h^−1^. The chemostats were sampled every other day, from the inoculation of a small number of *Microcystis* CYA140 cells to steady state with high population densities.

Next, we studied the steady states of six chemostats of *Microcystis* HUB5-2-4 along a gradient from carbon-limited to light-limited conditions. The chemostats had a dilution rate of *D* = 0.00625 h^−1^, and were provided with different pCO_2_ concentrations in the gas flow (0.5, 50, 100, 400 or 2,800 ppm CO_2_) and two different NaHCO_3_ concentrations in the mineral medium (0.5 or 2.0 mmol L^−1^). The steady states were monitored for at least ten days.

#### Measurements

The incident light intensity (*I_IN_*) and the light intensity transmitted through the chemostat vessel (*I_OUT_*) were measured with a LI-COR LI-250 quantum photometer (LI-COR Biosciences, Lincoln, NE, USA) at 10 randomly chosen positions on the front and back surface of the chemostat vessel, respectively. Background turbidity (*K_bg_*) was calculated from the light transmission through chemostat vessels without phytoplankton using Lambert-Beer’s law, as *K_bg_* = ln(*I_IN_*/*I_OUT_*)/*z_MAX_*.

DIC concentrations were determined by sampling 15 mL of culture suspension, which was immediately filtered over 0.45 µm membrane filters (Whatman, Maidstone, UK). DIC was subsequently analyzed by phosphoric acid addition on a Model 700 TOC Analyzer (OI Corporation, College Station, TX, USA), with a detection limit of 0.15 ppm. Temperature and pH were measured with a SCHOTT pH meter (SCHOTT AG, Mainz, Germany). Concentrations of dissolved CO_2_, bicarbonate and carbonate were calculated from DIC and pH [30], based on the dissociation constants of inorganic carbon corrected for temperature and salinity (Table S2.1 in Text S2). Alkalinity was determined in a 50 mL sample that was titrated in 0.1 to 1 mL steps with 10 mmol L^−1^ HCl to a pH of 3.0. The alkalinity was subsequently calculated using Gran plots [30].

Residual nitrate and phosphate concentrations in the chemostats were determined in triplicate by sampling 15 mL of culture suspension, which was immediately filtered over 0.45 µm membrane filters (Whatman, Maidstone, UK) and the filtrate was stored at −20°C. Nitrate concentrations were analyzed using a Skalar SA 400 autoanalyzer (Skalar Analytical B.V., Breda, The Netherlands), and phosphate concentrations were analyzed spectrophotometrically [59].

Phytoplankton population density, both as cell numbers and total biovolume, was determined in triplicate using a Casy 1 TTC cell counter with a 60 µm capillary (Schärfe System GmbH, Reutlingen, Germany). Cell size varied considerably during the experiments, ranging from 31–66 µm^3^ cell^−1^ in *Microcystis* CYA140 and from 25–50 µm^3^ cell^−1^ in *Microcystis* HUB5-2-4. We therefore used the total biovolume (i.e. the summed volume of all cells per litre of water) as a measure of phytoplankton population density.

Samples for cellular carbon, nitrogen, phosphorus and sulfur content were pressurized at 10 bar to collapse the gas vesicles of *Microcystis* and subsequently centrifuged for 15 min at 2,000 *g*. After discarding the supernatant, the pellet was resuspended in demineralised water, and centrifuged for 5 min at 15,000 *g*. The supernatant was discarded, pellets were stored at −20°C and subsequently freeze-dried and weighted to determine dry weight. The carbon, nitrogen and sulfur content of homogenised freeze-dried cell powder were analysed using a Vario EL Elemental Analyzer (Elementar Analysensysteme GmbH, Hanau, Germany). To determine the phosphorus content, cells were oxidized with potassium persulfate for 1 h at 100°C [60], and phosphate concentrations were subsequently analyzed spectrophotometrically [59].

To calculate the carbon sequestration rate of the experiments at steady state, we solved Eqns (1), (2) and (4) for zero. This yields:
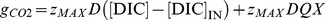
(8)where we assumed that the specific loss rate of the phytoplankton was governed by the dilution rate of the chemostat (i.e., *m* = *D*). This equation shows that, at steady state, the carbon sequestration rate equals the net enhancement of the DIC concentration plus the carbon fixation rate of the phytoplankton population.

### Parameter estimation

System parameters such as incident light intensity, mixing depth of the chemostats, composition of the mineral medium, dilution rate and CO_2_ concentration in the gas flow were measured prior to and/or during the experiments. Some phytoplankton parameters were measured experimentally, while others were estimated from fits of the model predictions to time courses of the experimental variables following the same procedures as in earlier studies [49], [55]. An overview of all parameter estimates is given in Text S2.

### Extrapolation to lakes

Chemostats provide ideal systems to test models under highly controlled conditions. They operate at the laboratory scale, with parameter settings tuned to the small size of the chemostat vessel. To extrapolate the model predictions to natural waters, we therefore adapted several model assumptions. Phytoplankton parameters were still based on our laboratory experiments with *Microcystis* HUB5-2-4. However, we used physical and chemical parameter settings typical for the summer situation in eutrophic lakes based on our data from Lake Volkerak, The Netherlands [31]. For instance, the mixing depth was increased from a chemostat of only 5 cm deep to a lake of 5 m deep. The very high phosphate and nitrate concentrations in the mineral medium of the chemostat were reduced to a lower (but still fairly high) phosphate concentration of 15 µmol L^−1^ and nitrate concentration of 150 µmol L^−1^, representative for hypertrophic lakes dominated by cyanobacterial blooms [15], [25], [31]–[33]. The high influx of CO_2_ gas into the chemostat vessel was replaced by a low gas transfer velocity across the air-water interface of lakes [54], [61], [62]. Full implementation of the lake model is described in Text S2 and Text S3.

### Sensitivity analysis

We performed a sensitivity analysis to assess how variation in the model parameters would affect the model predictions. In this analysis, we focus on low-alkaline lakes (ALK_IN_ = 0.5 mEq L^−1^), since they are more sensitive to rising atmospheric CO_2_ concentrations than high-alkaline lakes. The sensitivity analysis investigates how the model predictions were affected by variation in two input parameters: (i) the atmospheric CO_2_ level and (ii) a second model parameter of choice. In contrast to traditional one-factor-at-a-time (OAT) sensitivity analysis, this two-dimensional approach may reveal possible interactions between the two model parameters [63]. For instance, model predictions might be more sensitive to parameter changes at low than at high atmospheric CO_2_ levels.

In addition, we calculated the normalized sensitivity coefficient (*SC*), which is a local sensitivity index that quantifies the relative change in model output *Y* with respect to a relative change in input parameter *Z* [64]:
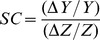
(9)


The normalized sensitivity coefficient is dimensionless, and allows comparison between input and output parameters independent of their units of measurement. |*SC*|>>1 implies that the model prediction is very sensitive to a change in the input parameter, whereas |*SC*|<<1 implies that the model prediction is rather insensitive to a change in the input parameter. We based the calculation of *SC* on a 1% increment of the input parameter. The sensitivity coefficient was calculated at two atmospheric CO_2_ levels, the present-day level of 400 ppm and an elevated level of 750 ppm predicted for the year 2150 by the RCP6 scenario of the Fifth Assessment Report of the IPCC [1].

## Results

### Dynamic changes during phytoplankton growth

**Figure 2:**
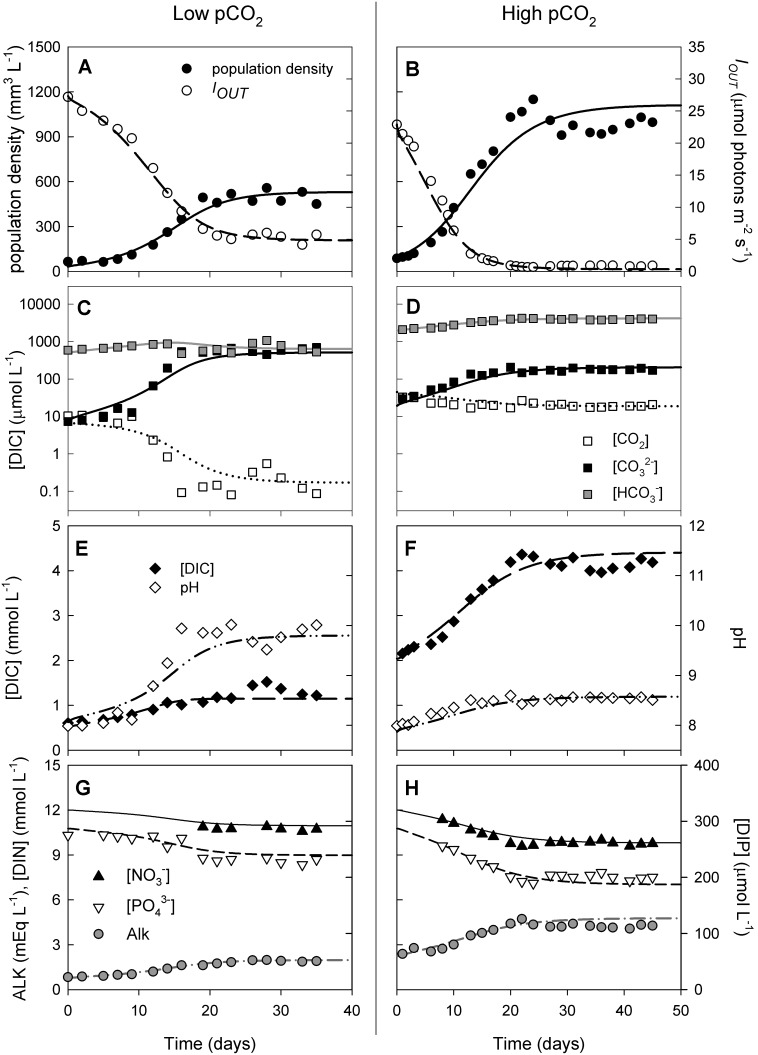
Changes in inorganic carbon chemistry during phytoplankton growth in two chemostat experiments. Left panels: Chemostat experiment with low pCO_2_ of 200 ppm in the gas flow and 500 µmol L^−1^ bicarbonate in the mineral medium. Right panels: Chemostat experiment with high pCO_2_ of 1,200 ppm in the gas flow and 2,000 µmol L^−1^ bicarbonate in the mineral medium. Both chemostats were inoculated with *Microcystis* CYA140. (A, B) Population density (expressed as biovolume) and light intensity penetrating through the chemostat (*I_OUT_*), (C, D) dissolved CO_2_, bicarbonate and carbonate concentrations, (E, F) total DIC concentration and pH, and (G, H) alkalinity (ALK) and concentrations of dissolved inorganic nitrogen (DIN) and phosphorus (DIP). Symbols represent measurements, lines show the model fits. The model and its parameter values are detailed in Text S2.

We studied dynamic changes in inorganic carbon chemistry during the growth of *Microcystis* CYA140 in two chemostats that differed with respect to the pCO_2_ level in the gas flow and the bicarbonate concentration in the medium (Fig. 2). In both chemostats, the population density increased after inoculation, while light penetration (*I_OUT_*) decreased due to shading by the growing *Microcystis* populations, until steady state was reached after ∼30 days (Figs. 2A and 2B). At high pCO_2_ the population density became two times higher and light penetration decreased more strongly than at low pCO_2_.

Phytoplankton growth impacted DIC, pH and alkalinity in both chemostats, but in a different way. With a low pCO_2_ in the gas flow, the growing phytoplankton population depleted the dissolved CO_2_ concentration over almost two orders of magnitude, from 10 to 0.2 µmol L^−1^, while the bicarbonate concentration varied between 600 and 900 µmol L^−1^ (Fig. 2C). At high pCO_2_, the dissolved CO_2_ concentration was much less depleted, while the bicarbonate concentration doubled from 2,000 µmol L^−1^ at inoculation to 4,000 µmol L^−1^ at steady state (Fig. 2D). The strong CO_2_ depletion raised the pH from 8 to 10 at low pCO_2_ (Fig. 2E), while the pH increased only to ∼8.5 at high pCO_2_ (Fig. 2F). The increase in pH mediated a shift in carbon speciation in both chemostats, although the shift was more dramatic at low pCO_2_ (Fig. 2C and 2D). In particular, the carbonate concentration increased to ∼45% of the total DIC at low pCO_2_, while it remained at only 4% at high pCO_2_. The total DIC concentration increased from 600 to 1,000 µmol L^−1^ at low pCO_2_ (Fig. 2E), and from 2,100 to 4,200 µmol L^−1^ at high pCO_2_ (Fig. 2F).

Despite the increase in total DIC, the phytoplankton experienced considerable carbon limitation (*L_C_* = 44%) in the experiment at low pCO_2_. This was primarily due to depletion of the dissolved CO_2_ concentration. Carbonate is unavailable for uptake, while our model estimated a half-saturation constant for bicarbonate of 75 µmol L^−1^ (Table S2.3 in Text S2), indicating that the bicarbonate uptake rate was essentially saturated with bicarbonate throughout the experiment. At high pCO_2_, carbon limitation was negligible (*L_C_* = 2%), and growth was primarily limited by the low availability of light. At steady state, the light intensity penetrating through the chemostat vessel (*I_OUT_*) was only 0.8 µmol photons m^−2 ^s^−1^ (Fig. 2B).

The growing phytoplankton population reduced the residual nitrate and phosphate concentration, yet nitrate and phosphate remained available at saturating concentrations of >10 mmol N L^−1^ and >180 µmol P L^−1^, respectively (Fig. 2G and 2H). Hence, nitrate and phosphate were not depleted to limiting levels. However, uptake of nitrate, phosphate and sulfate by phytoplankton consumed H^+^ ions and thereby increased alkalinity in both chemostats (Fig. 2G and 2H). Since a larger population density consumes more nutrients, alkalinity increased more strongly in the high pCO_2_ than in the low pCO_2_ treatment. The model fits captured the coupling between phytoplankton growth, carbon availability, nutrients, light, pH and alkalinity quite well at both low and high pCO_2_ levels (Fig. 2).

### Separation of time scales

Because of the relatively high dimensionality of our model, formal mathematical analysis of the existence, uniqueness and stability of the equilibrium point is not straightforward. Therefore, we explored the full phase space of the model by extensive numerical simulations. This did not reveal any indications for alternative stable states or non-equilibrium dynamics. Instead, we always found at most one unique positive equilibrium point that was locally and globally stable whenever it existed.

**Figure 3:**
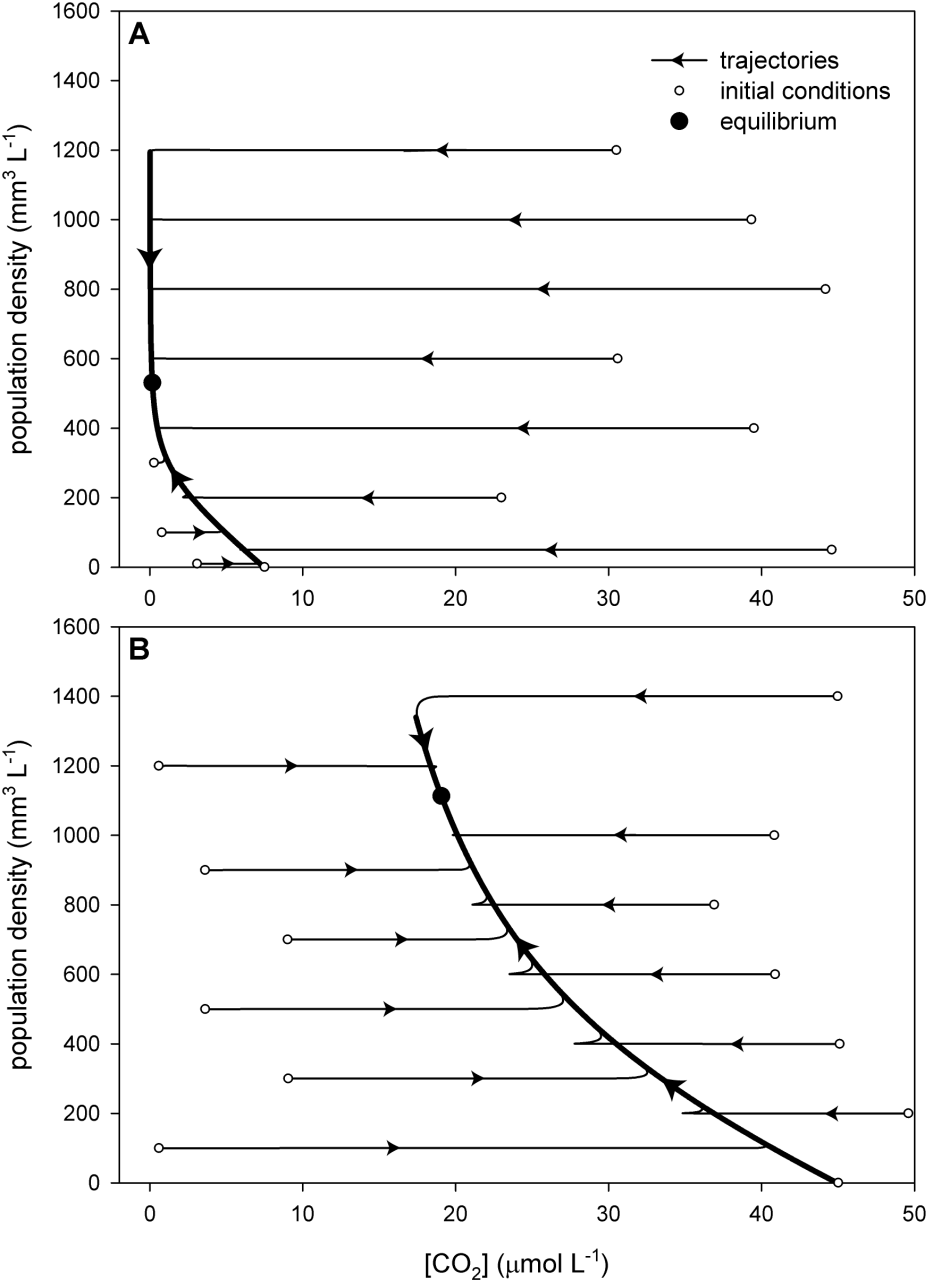
Trajectories of dissolved CO_2_ and population density. Trajectories predicted by the model for chemostats with (A) low pCO_2_ of 200 ppm in the gas flow and 500 µmol L^−1^ bicarbonate in the mineral medium, and (B) high pCO_2_ of 1,200 ppm in the gas flow and 2,000 µmol L^−1^ bicarbonate in the mineral medium. The trajectories start from a series of different initial conditions, and all converge to the same equilibrium point. Arrows indicate the direction of the trajectories. The model assumes species parameters specific for *Microcystis* CYA140, and is detailed in Text S2.

Two examples are given in Fig. 3, where we used the calibrated model to investigate trajectories of dissolved CO_2_ and population density from a range of different initial conditions. Interestingly, the trajectories show that the dynamics operated at two distinct time scales: fast chemical dynamics and slow biological dynamics. The inorganic carbon chemistry equilibrated with the standing population density within a few hours, as indicated by the horizontal parts of the trajectories in Fig. 3. These rapid dynamics are consistent with the inorganic carbon chemistry in our chemostat experiments without phytoplankton, which also equilibrated within 1–4 hours (Fig. S4.1 in Text S4). Subsequently, the population density slowly converged to equilibrium within a time span of several weeks. These slow dynamics are indicated in Fig. 3 by the thick curved parts of the trajectories, which ultimately lead to the equilibrium point. Hence, the inorganic carbon chemistry rapidly adjusted to the standing population, and subsequently tracked the slower changes in population density.

### Steady-state patterns

**Figure 4:**
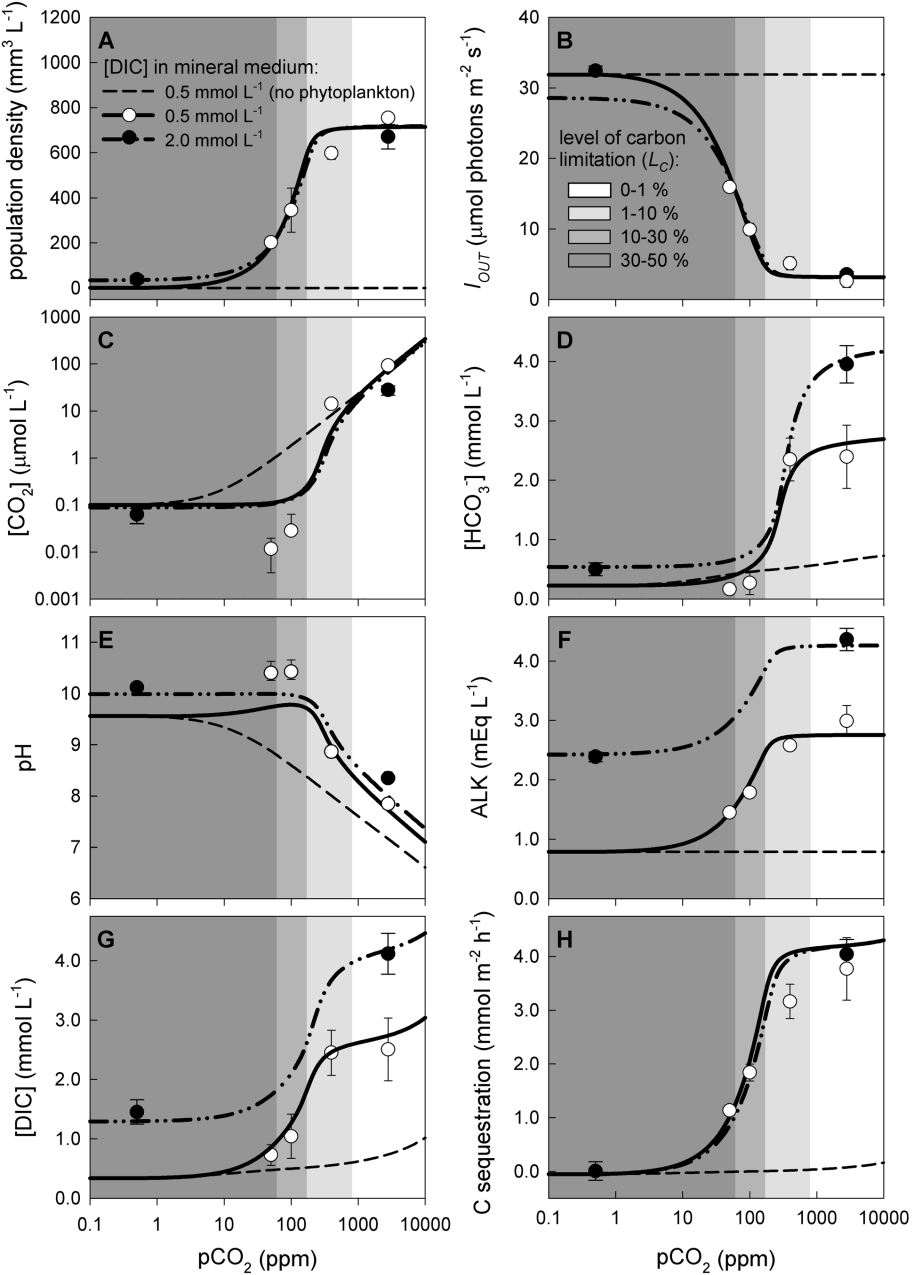
Steady-state patterns of phytoplankton population density and inorganic carbon chemistry in chemostat experiments. Steady-state results are shown for 6 chemostats with *Microcystis* HUB5-2-4 exposed to different pCO_2_ levels in the gas flow and two different bicarbonate concentrations in the mineral medium (0.5 or 2.0 mmol L^−1^). (A) Phytoplankton population density (expressed as biovolume), (B) light intensity penetrating through the chemostat (*I_OUT_*), (C) dissolved CO_2_ concentration, (D) bicarbonate concentration, (E) pH, (F) alkalinity, (G) DIC concentration, and (H) carbon sequestration rate. Symbols show the mean (± s.d.) of 5 measurements in each steady-state chemostat, lines show the model fits. For comparison, dashed lines show steady-state patterns predicted for chemostats without phytoplankton. Shading indicates the level of carbon limitation (*L_C_*) predicted by the model. The model and its parameter values are detailed in Text S2.

We investigated steady-state patterns of phytoplankton abundance and inorganic carbon chemistry using six chemostats of *Microcystis* HUB5-2-4 (Fig. 4). The steady-state population density increased with pCO_2_, demonstrating that it was limited by the supply of inorganic carbon. The population density leveled off when carbon limitation was alleviated at pCO_2_>200 ppm (Fig. 4A). At pCO_2_ levels <1 ppm, a low DIC concentration of 0.5 mmol L^−1^ in the mineral medium provided insufficient inorganic carbon, whereas a higher DIC concentration of 2.0 mmol L^−1^ was sufficient to sustain a steady-state population density. At pCO_2_ levels >100 ppm, the influx of DIC supplied by the mineral medium was small compared to the influx of CO_2_ supplied by the high gas flow rate, such that the four-fold difference in DIC concentration in the mineral medium had little effect on the steady-state population density.

The increase in population density with rising pCO_2_ reduced light penetration through the chemostats (Fig. 4B), which shifted the growth conditions from carbon limitation at low pCO_2_ to light limitation at high pCO_2_. At pCO_2_ levels <100 ppm, phytoplankton strongly depleted the dissolved CO_2_ concentration to a stable level of ∼0.1 µmol L^−1^ (Fig. 4C), while pH was maintained at values around 10 (Fig. 4E). At pCO_2_ levels >100 ppm, the dissolved CO_2_ concentration increased and pH decreased with increasing pCO_2_ (Fig. 4C, E). The pH remained consistently higher in the presence than in the absence of phytoplankton.

Counterintuitively, at pCO_2_>100 ppm, the bicarbonate concentration became higher in the presence than in the absence of phytoplankton (Fig. 4D), even though phytoplankton consume bicarbonate as inorganic carbon source. This unexpected result is caused by the shift in pH in combination with an increase in alkalinity associated with uptake of nitrate, phosphate and sulfate by the phytoplankton population (Fig. 4F; see also Eqn (7)). An increased alkalinity enhances the storage capacity for bicarbonate and carbonate in the system. The alkalinity, bicarbonate concentration and total DIC concentration all showed a similar increase with rising pCO_2_ as the phytoplankton population density (compare Figs. 4D, F, G with Fig. 4A). At pCO_2_>200 ppm, 70–80% of the total amount of carbon in the system was in phytoplankton biomass while 20–30% of the total carbon was DIC.

The carbon sequestration rate also showed a similar increase with rising pCO_2_ as the DIC concentration and phytoplankton population density, and leveled off when the population approached its maximum productivity at >200 ppm (Fig. 4H).

The model fits were in good agreement with the observed steady-state patterns in phytoplankton population density, inorganic carbon availability, alkalinity and pH along the entire CO_2_ gradient.

### Extrapolation to lakes

**Figure 5:**
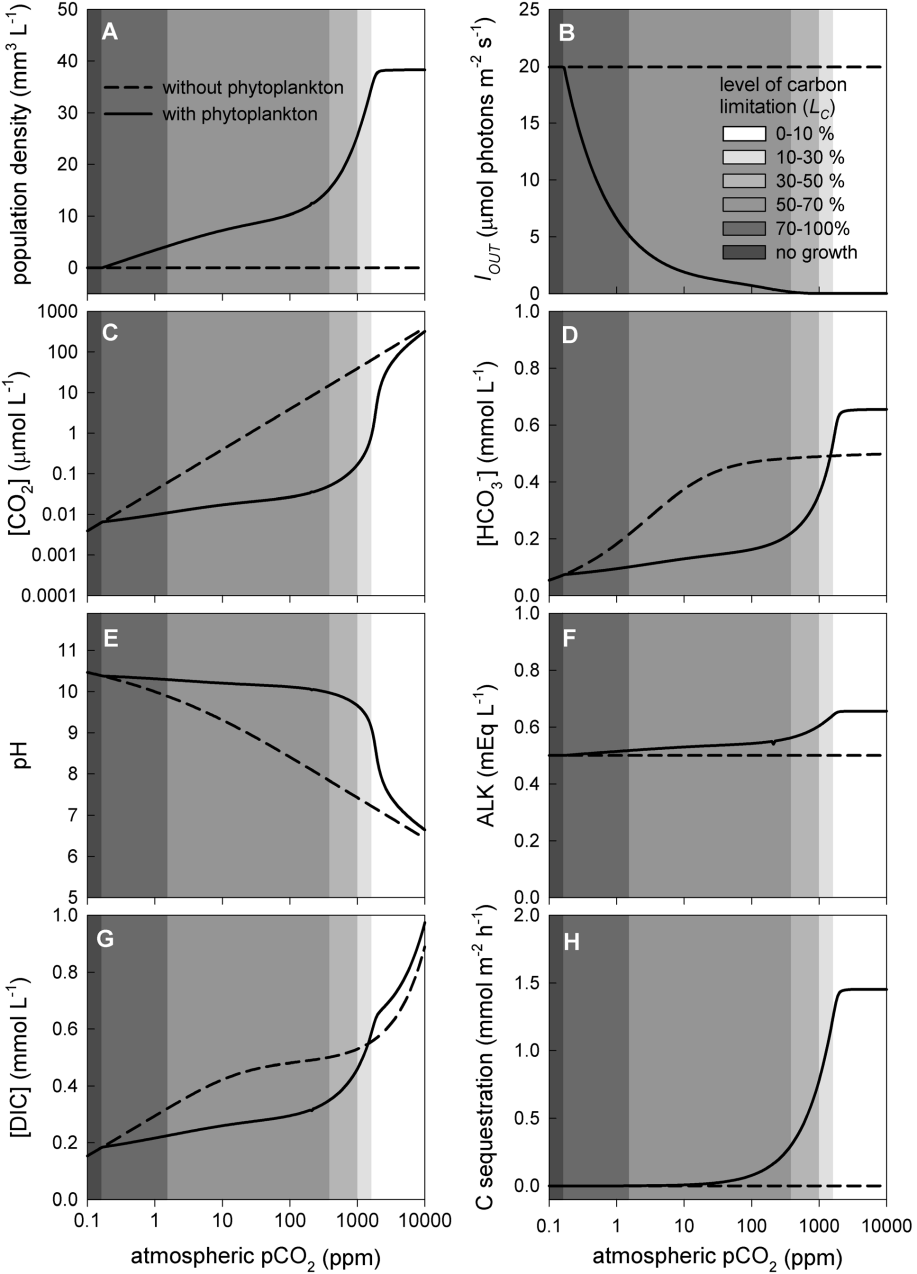
Steady-state patterns predicted for phytoplankton blooms in low-alkaline lakes. Steady-state predictions of the model evaluated across a wide range of atmospheric pCO_2_ levels. (A) Phytoplankton population density (expressed as biovolume), (B) light intensity reaching the lake sediment (*I_OUT_*), (C) dissolved CO_2_ concentration, (D) bicarbonate concentration, (E) pH, (F) alkalinity, (G) DIC concentration, and (H) carbon sequestration rate. Shading indicates the level of carbon limitation (*L_C_*). For comparison, dashed lines show steady-state patterns predicted for low-alkaline waters without phytoplankton. The model parameters are representative for eutrophic low-alkaline lakes (ALK_IN_ = 0.5 mEq L^−1^) dominated by the cyanobacterium *Microcystis* HUB5-2-4. The model and its parameter values are detailed in Text S2 and Text S3.

The model was adapted to natural waters to explore the impact of rising atmospheric CO_2_ levels on phytoplankton blooms in lakes. Although it is difficult to capture the complex dynamics of natural systems, such a modelling exercise may help in understanding the coupling between phytoplankton blooms and inorganic carbon chemistry. As a first step, we investigated steady-state patterns of phytoplankton abundance in low-alkaline lakes, where bicarbonate concentrations are low and phytoplankton growth therefore largely depends on dissolved CO_2_ as a carbon source. This is a similar situation as in our chemostat experiments, and the model predictions for low-alkaline lakes are therefore qualitatively similar to the results obtained in our chemostats (compare Fig. 4 and Fig. 5). The phytoplankton population can be sustained at pCO_2_ levels above 0.17 ppm, and is predicted to increase strongly with pCO_2_ (Fig. 5A). Above 2,000 ppm, a further rise of the pCO_2_ level no longer enhances the population density, because the high CO_2_ supply in combination with self-shading in dense phytoplankton blooms has shifted phytoplankton growth from carbon-limited to light-limited conditions (Fig. 5B).

Over a wide range of pCO_2_ levels, from 0.17 to 1,000 ppm, phytoplankton blooms exert strong control over the dissolved CO_2_ concentration and pH, depleting the dissolved CO_2_ concentration below 0.1 µmol L^−1^ and raising pH to 10 (Fig. 5C, E). The bicarbonate and total DIC concentration are reduced by the phytoplankton population for pCO_2_ levels ranging from 0.17 to 1,400 ppm CO_2_ (Fig. 5D, G). The bicarbonate concentration, total DIC concentration, alkalinity and carbon sequestration rate all increase with rising pCO_2_, and level off when the phytoplankton population approaches maximum densities (Fig. 5D, F–H). Above 1,000 ppm, phytoplankton blooms exert less control over CO_2_ availability and pH, and the dissolved CO_2_ concentration increases while pH decreases with a further rise in pCO_2_ (Fig. 5C, E).

**Figure 6:**
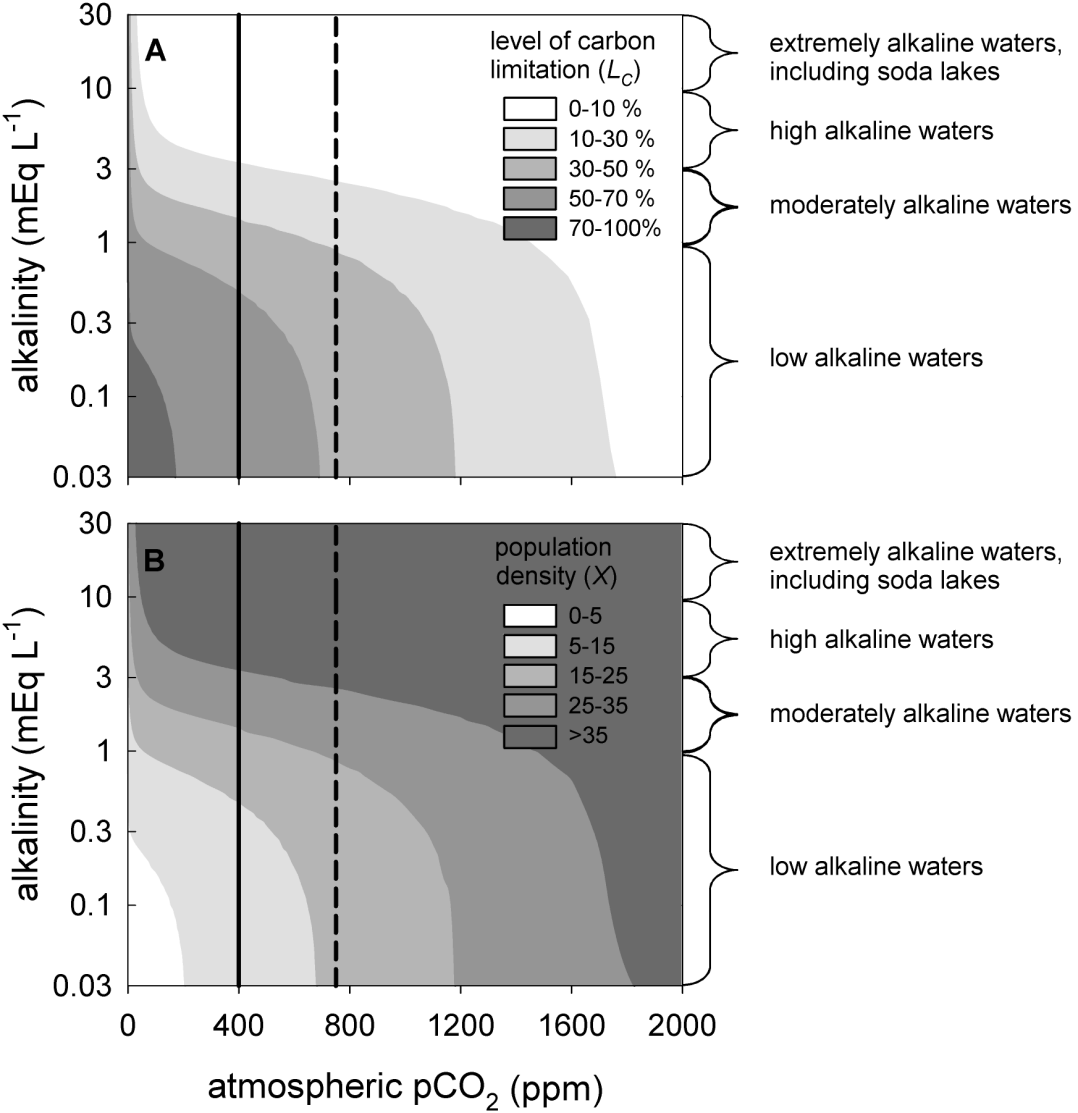
Contour plots of phytoplankton blooms predicted for different pCO_2_ levels and alkalinities. Model predictions of (A) the level of carbon limitation, and (B) phytoplankton population density (expressed as biovolume, in mm^3 ^L^−1^). The vertical solid line represents the present-day atmospheric CO_2_ level of ∼400 ppm, while the vertical dashed line shows the atmospheric CO_2_ level of 750 ppm predicted for the year 2150 by the RCP6 scenario of the Fifth Assessment Report of the IPCC. The model predictions are based on steady-state solutions across a grid of 40×50 = 2,000 simulations, using the model and parameter values detailed in Text S2 and Text S3.

Figure 6 summarizes the level of carbon limitation and the population density predicted for dense phytoplankton blooms in different eutrophic waters spanning a wide range of alkalinities and pCO_2_ levels. In line with expectation, the model predicts that carbon limitation of dense phytoplankton blooms will be most pronounced in low-alkaline waters, where CO_2_ provides the main inorganic carbon source (Fig. 6A). Rising atmospheric pCO_2_ levels are expected to lead to a strong increase in phytoplankton population density in these low-alkaline waters (Fig. 6B). In lakes with a moderate alkalinity, where bicarbonate can partially supplement growth when CO_2_ is depleted, carbon limitation is predicted to be less intense but may still play a substantial role (i.e., *L_C_* = 10–50%; Fig. 6A). In high-alkaline waters and soda lakes, however, carbon will rarely be limiting at ambient atmospheric pCO_2_ levels (Fig. 6A). Their large DIC pools provide a sufficient supply of CO_2_ and bicarbonate to produce high phytoplankton population densities at ambient pCO_2_ levels (Fig. 6B).

### Sensitivity analysis

#### Phytoplankton traits

**Figure 7:**
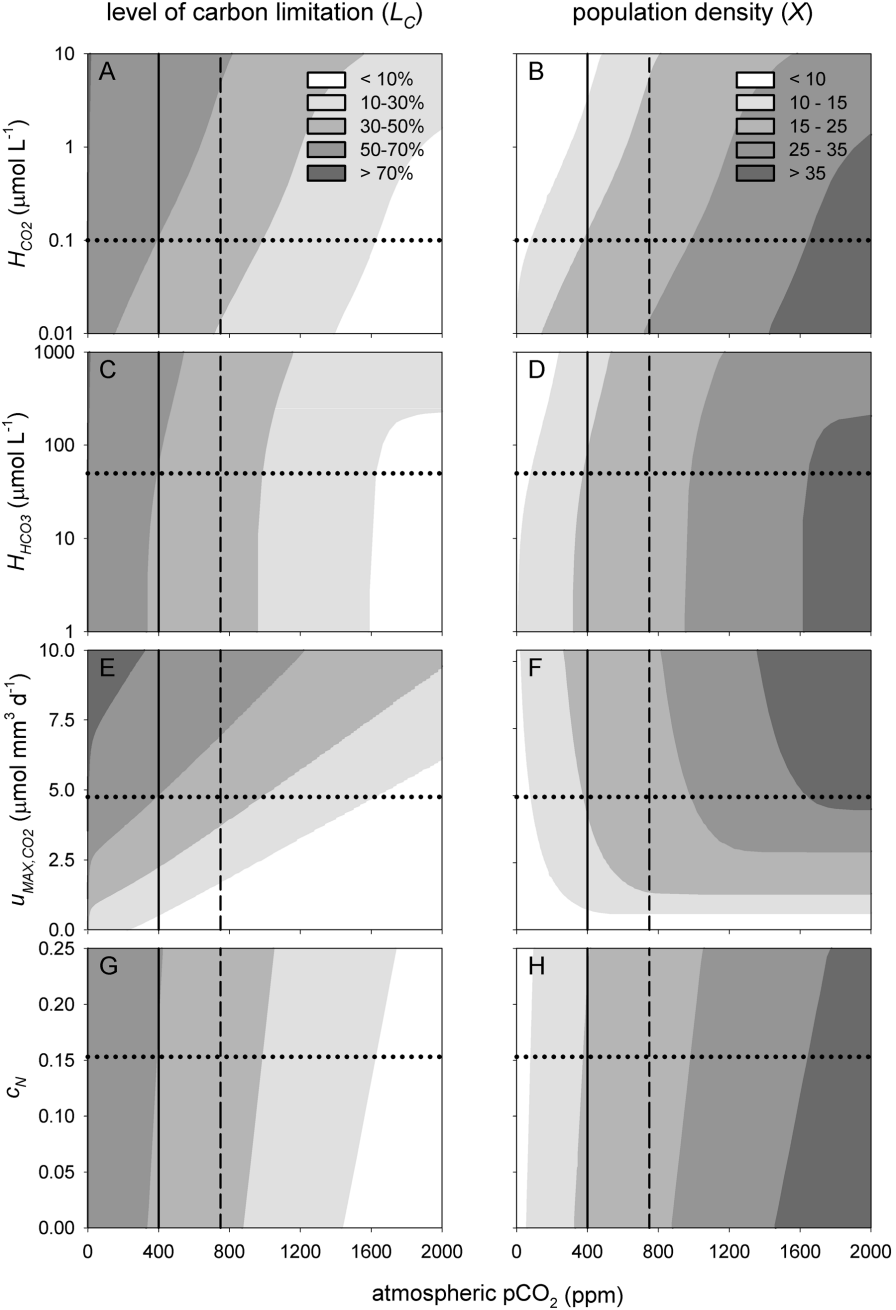
Sensitivity of the model predictions to variation in phytoplankton traits. Contour plots of the level of carbon limitation (left panels) and steady-state phytoplankton population density (right panels, expressed as biovolume, in mm^3 ^L^−1^) predicted for different atmospheric pCO_2_ levels and phytoplankton traits. The phytoplankton traits are (A, B) the half-saturation constant for CO_2_ uptake (*H_CO2_*), (C, D) the half-saturation constant for bicarbonate uptake (*H_HCO3_*), (E, F) the maximum CO_2_ uptake rate (*u_MAX, CO2_*), and (G, H) the cellular N:C ratio (*c_N_*). The model considers a low-alkaline lake (*ALK_IN_* = 0.5 mEq L^−1^). Vertical lines represent atmospheric CO_2_ levels of 400 ppm (present-day) and 750 ppm (predicted for the year 2150 by the RCP6 scenario of the IPCC). Horizontal dotted lines represent our default parameter values. The contour plots are based on steady-state solutions across a grid of 40×50 = 2,000 simulations.

As a first step, we investigated the sensitivity of the model predictions to variation in the half-saturation constant for CO_2_ uptake (Fig. 7A, B). Note that an increase of the half-saturation constant implies a reduced affinity. All else being equal, an increase in the half-saturation constant for CO_2_ therefore leads to stronger carbon limitation and lower phytoplankton population densities (Fig. 7A, B). The normalized sensitivity coefficients were small, both at 400 and at 750 ppm (Table 1). A value of *SC* = 0.10 implies that for a 1% increase in the half-saturation constant, the model predicts only a 0.1% increase in the level of carbon limitation. Hence, the sensitivity of the model predictions to variation in the half-saturation constant for CO_2_ uptake is relatively low.

**Table 1:** Normalized sensitivity coefficients of selected model parameters at atmospheric CO_2_ levels of 400 ppm (*SC*_400_) and 750 ppm (*SC*_750_).

Parameter	Description	Level of carbon limitation		Population density	
		*SC_400_*	*SC_750_*	*SC_400_*	*SC_750_*
Species traits					
*H_CO2_*	Half-saturation constant for CO_2_ uptake	0.07	0.10	–0.11	–0.09
*H_HCO3_*	Half-saturation constant for bicarbonate uptake	0.03	0.03	–0.04	–0.02
*u_MAX, CO2_*	Maximum uptake rate of CO_2_	0.56	0.88	0.17	0.17
*c_N_*	Cellular N:C ratio	0.04	0.08	–0.06	–0.07
Lake properties					
*z_MAX_*	Lake depth	–0.51	–0.65	–0.78	–0.87
*v*	Gas transfer velocity of CO_2_	–0.25	–0.63	0.40	0.54
[DIC]_IN_	Concentration of DIC at influx	–0.61	–0.84	0.96	0.71
*Sal*	Salinity	0.00	–0.01	0.00	0.01

The normalized sensitivity coefficient expresses the relative change in model output with respect to a relative change in input parameter. We used several species traits and lake properties as input parameters, and the level of carbon limitation and phytoplankton population density as model output.

The half-saturation constant for bicarbonate shows a similar pattern (Fig. 7C, D).

An increase in the maximum uptake rate of CO_2_ causes stronger CO_2_ depletion during phytoplankton blooms, which results in stronger carbon limitation and higher population densities (Fig. 7E, F). Interestingly, comparison of the sensitivity coefficients indicates that changes in the maximum uptake rate of CO_2_ have a larger effect on the level of carbon limitation than on the phytoplankton population density (Table 1).

Changes in the C:N stoichiometry of phytoplankton cells do not directly affect the growth rates in our model, because we assumed that all nutrients are available at saturating levels. Changes in cellular C:N stoichiometry may have a small indirect effect, however, because nitrate uptake affects alkalinity, and thereby inorganic carbon availability. Hence, as expected, the model predictions are rather insensitive to changes in cellular C:N stoichiometry (Fig. 7G, H; Table 1).

#### Lake properties

**Figure 8:**
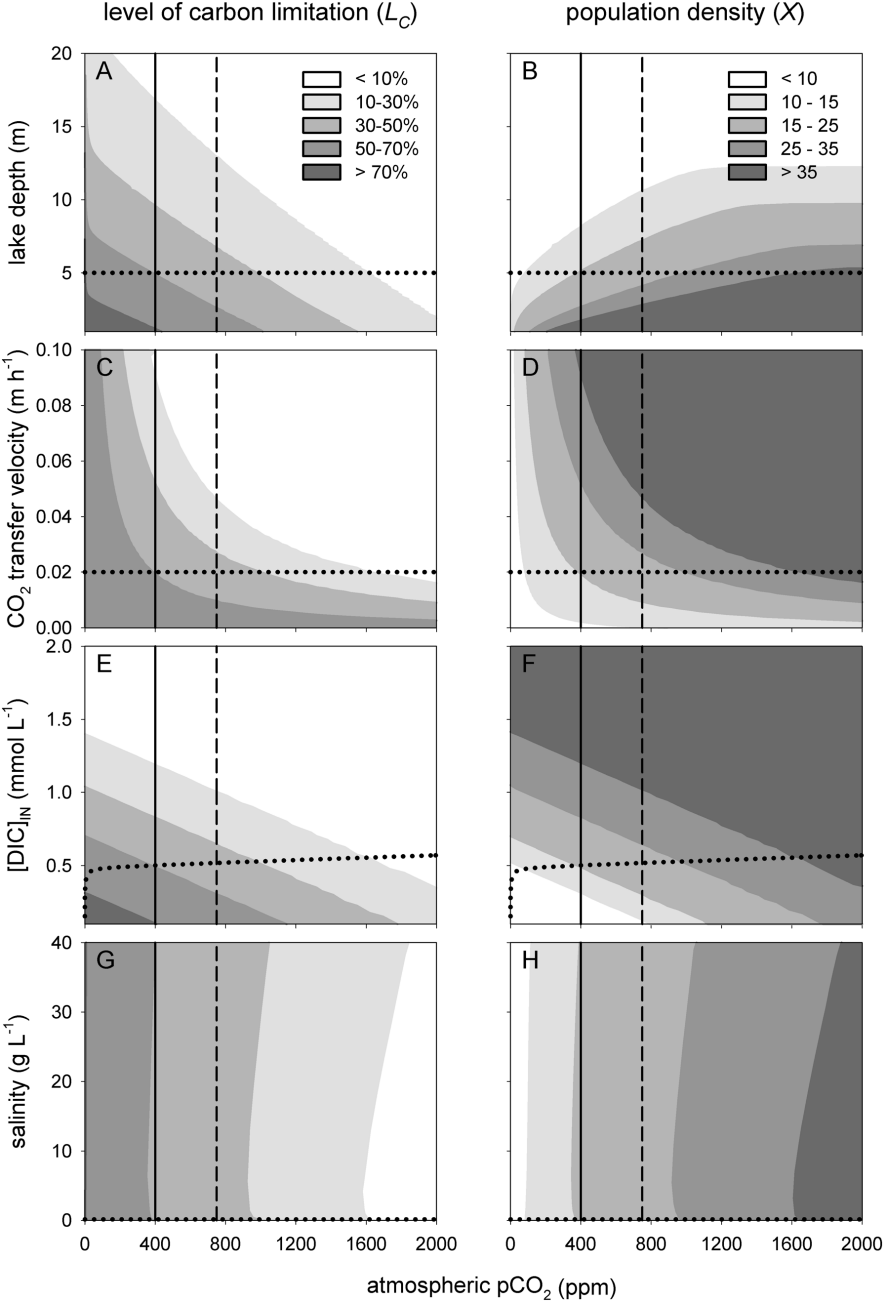
Sensitivity of the model predictions to variation in lake properties. Contour plots of the level of carbon limitation (left panels) and steady-state phytoplankton population density (right panels, expressed as biovolume, in mm^3 ^L^−1^) predicted for different atmospheric pCO_2_ levels and lake properties. The lake properties are (A, B) lake depth (*z_MAX_*), (C, D) CO_2_ gas transfer velocity (*v*), (E, F) DIC concentration of the influx ([DIC]_IN_), and (G, H) salinity (*Sal*). The model considers a low-alkaline lake (*ALK_IN_* = 0.5 mEq L^−1^). Vertical lines represent atmospheric CO_2_ levels of 400 ppm (present-day) and 750 ppm (predicted for the year 2150 by the RCP6 scenario of the IPCC). Horizontal dotted lines represent our default parameter values. In (E, F), the dotted line indicates equilibrium with the atmospheric CO_2_ pressure. The contour plots are based on steady-state solutions across a grid of 40×50 = 2,000 simulations.

Lake depth has strong effects on the model predictions. In deep lakes, the phytoplankton population is spread out over a large water volume, and will be light-limited in deeper parts of the water column. Hence, all else being equal, CO_2_ depletion in deep lakes will be less intense, resulting in lower levels of carbon limitation than in shallow lakes (Fig. 8A). Phytoplankton population densities are therefore predicted to respond more strongly to rising pCO_2_ levels in shallow than in deep lakes (Fig. 8B).

The CO_2_ gas transfer velocity across the air-water interface varies with wind speed and precipitation events [54], [61], [62]. An increase in CO_2_ gas transfer velocity strongly reduces the level of carbon limitation and increases the phytoplankton population density (Fig. 8C, D). Interestingly, the sensitivity coefficients point at an interactive effect with the atmospheric CO_2_ level. The model predictions become more sensitive to changes in CO_2_ gas transfer velocity at higher atmospheric CO_2_ levels (Table 1).

Enhanced mineralization of organic carbon in the sediment or additional CO_2_ input from the surrounding watershed may cause an enhanced CO_2_ influx into the lake. In our model this would be represented by an increase in DIC influx without a change in alkalinity. Such an enhanced CO_2_ influx reduces the level of carbon limitation, thereby raising phytoplankton population density (Fig. 8E, F). The sensitivity coefficients indicate that the model predictions respond strongly to changes in DIC input (Table 1).

Salinity has a negative impact on the solubility of CO_2_ in water [65], but a positive impact on the dissociation constants of carbonic acid and bicarbonate [66]. We explored salinities from 0 to 40 g L^−1^, covering the full salinity range from freshwater lakes to the oceans. The results show that, all else being equal, changes in salinity have only minor effects on the predicted level of carbon limitation and phytoplankton population density (Fig. 8G, H; Table 1).

All normalized sensitivity coefficients remained below 1, indicating that none of the model parameters had an unexpectedly strong nonlinear effect on the model output.

## Discussion

### Coupling between phytoplankton blooms and inorganic carbon chemistry

Our theoretical and experimental results demonstrate that the development of dense algal blooms can dramatically change the dissolved CO_2_ concentration, alkalinity and pH of aquatic ecosystems. In our experiments, phytoplankton growth induced a strong CO_2_ drawdown, especially when provided with a low pCO_2_ level in the gas flow. Assimilation of CO_2_ and nutrients such as nitrate, phosphate and sulfate increased alkalinity and pH during bloom development [38]–[40]. Increases in pH and alkalinity shifted the inorganic carbon composition towards bicarbonate and carbonate. These findings are in good agreement with field observations, as similar changes in DIC speciation, pH, and alkalinity have also been documented in studies of dense phytoplankton blooms in natural waters (Fig. 1) [11], [12], [15].

Dense phytoplankton blooms contribute to both ‘biological enhancement’ and ‘chemical enhancement’ of the CO_2_ influx into aquatic ecosystems. Biological enhancement is due to the drawdown of the dissolved CO_2_ concentration by dense phytoplankton blooms, which enlarges the CO_2_ concentration gradient across the air-water interface. Hence, dense phytoplankton blooms can turn aquatic ecosystems into net carbon sinks, and the resultant influx of atmospheric CO_2_ can further fuel phytoplankton growth [15], [67]. Chemical enhancement occurs because part of the influx of CO_2_ chemically reacts with water, and is transferred to bicarbonate and carbonate [68]. This chemical enhancement is promoted by the high pH and alkalinity induced by phytoplankton blooms, which enlarge the DIC storage capacity of aquatic ecosystems.

Interestingly, our laboratory experiments show that the enhanced CO_2_ influx induced by dense phytoplankton populations can even raise the bicarbonate and total DIC concentration (Fig. 2E, 2F, 4G). This may seem counterintuitive, because phytoplankton populations consume inorganic carbon. However, the high pH and alkalinity in phytoplankton blooms favors the formation of bicarbonate and carbonate. Depending on the interplay between CO_2_ gas transfer, inorganic carbon uptake, alkalinity and pH, this can result in either a decrease or increase in total DIC concentration. The lake model predicts that dense phytoplankton blooms may increase the bicarbonate and DIC concentration in lakes, but only at very high pCO_2_ levels. At pCO_2_ levels below 1,400 ppm, the lake model predicts a reduced bicarbonate and DIC concentration during phytoplankton blooms (Fig. 5D, G), which is supported by our observations from Lake Volkerak (Fig. 1B).

### Carbon limitation

In contrast to nutrients and light, carbon availability is often dismissed as an important limiting factor for phytoplankton growth. One common argument is that the CO_2_ concentrations in many freshwater lakes are sufficiently high to cover the carbon demands of phytoplankton populations, because these lakes are often supersaturated with CO_2_ [5], [6], [69]. However, dense phytoplankton blooms can strip surface waters from dissolved CO_2_, as has been observed in a wide range of aquatic ecosystems [11], [13], [15]. This is exemplified by our data from Lake Volkerak, which is supersaturated with CO_2_ in winter, yet dense cyanobacterial blooms deplete the CO_2_ concentration during the summer period (Fig. 1). Our laboratory experiments and model simulations indicate that dense phytoplankton blooms can deplete the dissolved CO_2_ concentration of low-alkaline waters by two to three orders of magnitude (Figs. 2, 4, 5).

Another common argument is that alkaline lakes typically have sufficiently high bicarbonate concentrations to cover the carbon demands of phytoplankton populations. Indeed, in addition to CO_2_, many phytoplankton species also utilize bicarbonate as carbon source [8]–[10]. However, utilization of bicarbonate requires additional investments in, e.g., sodium-dependent and ATP-dependent bicarbonate uptake systems and carbonic anhydrases [9], [10]. The costs of bicarbonate utilization may therefore have repercussions for the growth rates that can be achieved. *Synechococcus leopoliensis*, for instance, grows at ∼80% of its maximum growth rate when provided with bicarbonate as its main carbon source [70]. Our parameter estimates indicate that *Microcystis* CYA 140 grows at <50% while *Microcystis* HUB5-2-4 can only grow at 35% of its maximum growth rate on bicarbonate alone (Table S2.3 in Text S2). This is supported by the chemostat experiments. For instance, *Microcystis* HUB5-2-4 could just barely sustain a low population density when CO_2_ was largely removed from the gas flow, even though bicarbonate was provided at a saturating concentration of 2,000 µmol L^−1^ in the mineral medium (see the datapoint at 0.5 ppm pCO_2_ in Fig. 4A). For both strains, an increase in pCO_2_ level led to a clear increase in population density (Figs. 2 and 4). Hence, our experiments demonstrate that, even for cyanobacteria with their sophisticated carbon-concentrating mechanisms, increasing pCO_2_ levels in bicarbonate-rich waters can cause an increase in phytoplankton population density.

In line with expectation, our model predicts that the potential for carbon limitation strongly depends on alkalinity (Fig. 6). This is consistent with studies in natural waters. Carbon limitation is often observed during algal blooms in eutrophic low-alkaline lakes, where CO_2_ is the main inorganic carbon source [11], [71]. Carbon limitation has also been reported for moderately alkaline lakes (Fig. 1) [12], [13], [72], where bicarbonate partially supplements phytoplankton growth when CO_2_ is depleted. The model predicts that carbon limitation will be almost absent in high-alkaline waters and soda lakes, owing to their high inorganic carbon availability (Fig. 6). Indeed, tropical soda lakes are widely recognized to be among the world’s most productive ecosystems, and can sustain extremely dense populations of cyanobacteria [73], [74].

Only high nutrient loads can sustain phytoplankton blooms dense enough to deplete the dissolved CO_2_ concentration and induce carbon limitation [75]. In an analysis of 131 eutrophic lakes in the Midwestern USA, Balmer and Downing [15] showed that dissolved CO_2_ decreased below atmospheric equilibrium when total phosphorus (TP) concentrations exceeded 1–2 µmol L^−1^ and chlorophyll *a* levels exceeded 10–20 µg L^−1^. Severe CO_2_ depletion occurred at chlorophyll concentrations exceeding 80–100 µg L^−1^. This matches our data from Lake Volkerak, which has a summer TP concentration of ∼3 µmol L^−1^ [31], and where the dissolved CO_2_ concentration became undersaturated at chlorophyll concentrations exceeding 20 µg L^−1^ and was severely depleted during the height of the blooms (Fig. 1A). Such conditions also seem to be representative of several other eutrophic and hypertrophic lakes with dense phytoplankton blooms. For example, TP concentrations exceeding 2 µmol L^−1^ are also found in Lake Taihu in China [25], Lake Victoria in East Africa [76], the western part of Lake Erie, USA [23], [77], the southern part of Lake Peipsi on the border of Estonia and Russia [78], [79], and several smaller lakes and reservoirs [33], [71], [80], all of which have suffered from dense cyanobacterial blooms in summer. This indicates that the nutrient availability in these eutrophic and hypertrophic lakes is, at least in potential, high enough for dense phytoplankton blooms to induce carbon-limited conditions.

### Model limitations

Combining models and experiments has several advantages. It allows quantitative analysis of the different processes under controlled conditions. Furthermore, it ensures that model predictions are strongly grounded in measured data, which adds confidence to the model output. Moreover, the model aids interpretation of the experimental results, and also offers a tool for extrapolation of the investigated processes to natural waters (Figs. 4–7).

Nevertheless, like all models, our model is at best a major simplification of reality, based on a series of simplifying assumptions that ignore many of the intriguing complexities of the natural world. In particular, the domain of applicability of our model predictions is restricted to eutrophic and hypertrophic waters where all nutrients are in excess. In oligotrophic waters, rising atmospheric CO_2_ levels will probably have a much smaller effect on the development of phytoplankton blooms, because nutrient limitation suppresses phytoplankton growth [25], [81]. Therefore, we recently extended our model and associated experiments to nutrient-limited conditions [75]. This confirmed that, at low nutrient levels, rising CO_2_ concentrations will have much less impact on phytoplankton biomass development. Lower phytoplankton population densities will lead to less CO_2_ depletion and have a smaller impact on pH. However, at low nutrient levels, rising CO_2_ concentrations may lead to a strong increase of the carbon:nutrient stoichiometry of phytoplankton, with possible repercussions for their nutritional quality as food for herbivores [75].

Furthermore, natural waters vary in phytoplankton species composition, while our laboratory experiments were based on a single species only. Cyanobacteria and eukaryotic phytoplankton show genetic and physiological variation in carbon concentrating mechanisms, both between and within species [8]–[10]. Likewise, lakes vary in lake depth and DIC input from the surrounding watershed. We therefore performed a sensitivity analysis to assess how intrinsic uncertainty and natural variation in the model parameters would affect the model predictions. The sensitivity analysis shows that the model predictions are relatively robust to variation in species traits such as the half-saturation constants of CO_2_ and bicarbonate uptake, but respond strongly to changes in lake depth, CO_2_ gas transfer velocity and DIC input. The sensitivity of the model predictions to these lake properties indicates that the response to rising CO_2_ levels is likely to be lake specific. Yet, the general patterns predicted by the model are qualitatively robust, in the sense that rising atmospheric CO_2_ levels are predicted to alleviate the level of carbon limitation and to increase phytoplankton population densities irrespective of the exact parameter values used (Figs. 7, 8).

Finally, we emphasize that the extrapolation of our model to natural waters is intended to be of an exploratory nature. We focused exclusively on feedbacks between dense phytoplankton blooms and the inorganic carbon chemistry of lakes. However, many other processes are also known to affect phytoplankton blooms, such as nutrient availability, thermal stratification, and day-to-day weather variability [33], [82], [83]. Interactions with other species in the food web may induce phytoplankton-zooplankton oscillations [84], [85], the collapse of phytoplankton blooms by viruses [86], and other nonequilibrium dynamics [87]. Furthermore, the alkalinity and pH of natural systems is influenced not only by phytoplankton growth, but also by a variety of hydrological and biogeochemical processes [40], [42], [88]. Application of our model to specific phytoplankton blooms in specific lakes is encouraged, but will require incorporation of the myriad of additional processes that are considered to be of relevance for the particular lake under study.

### Effects of rising CO_2_

Our results support earlier reports that rising atmospheric pCO_2_ levels are likely to promote phytoplankton productivity in eutrophic waters [89], [90]. More specifically, both our model predictions and laboratory experiments indicate that elevation of the atmospheric pCO_2_ will enhance the CO_2_ influx across the air-water interface during dense phytoplankton blooms, which in turn will tend to further increase the population density of these blooms.

Our study shows that dense phytoplankton blooms are capable to deplete the dissolved CO_2_ concentration and increase the pH over a relatively wide range of atmospheric CO_2_ levels (Fig. 4C, E, Fig. 5C, E). Yet, at some point, rising atmospheric CO_2_ levels will alleviate phytoplankton blooms from carbon limitation, such that they will exert much less control over the dissolved CO_2_ concentration and pH. Beyond this point, phytoplankton blooms become carbon-saturated and a further rise in atmospheric CO_2_ levels will not enhance population densities but will lead to less intense CO_2_ depletion and a lower pH during the blooms. Our results indicate that the atmospheric CO_2_ level at which blooms become carbon-saturated is likely to vary among phytoplankton species depending on e.g. their carbon concentrating mechanisms [8]–[10], and among ecosystems depending on e.g. alkalinity, lake depth and CO_2_ input from the mineralization of dissolved organic carbon (Figs. 6–8). For instance, in our chemostat experiments the population density increased for atmospheric pCO_2_ levels from 10 to 200 ppm, while the *Microcystis* population became carbon-saturated at pCO_2_ levels beyond 200 ppm (Fig. 4A). In contrast, for low-alkaline lakes, our baseline model predicts a strong increase in phytoplankton population density from 100 to 1,500 ppm, while the transition to carbon-saturated *Microcystis* blooms is predicted to occur only when the pCO_2_ level exceeds 1,500 ppm (Fig. 5A).

Intensification of phytoplankton blooms by rising CO_2_ levels may further aggravate the problems associated with these blooms, such as anoxia and fish kills [82], [91] and the loss of submerged vegetation due to an increased turbidity [83], [84]. In particular, enhanced bloom formation by toxic phytoplankton species, like the cyanobacterium *Microcystis* of this study, can pose health risks for humans and animals, and may threaten the use of eutrophic waters for recreation, fisheries, drinking water and agricultural irrigation [21]–[25].

## Conclusions

The interplay between data-driven models and model-driven experimentation explored by our study may offer an important step towards an improved quantitative understanding and prediction of the impacts of rising CO_2_ on phytoplankton blooms. Our results demonstrate that, under controlled laboratory conditions, the coupling between phytoplankton growth, CO_2_ drawdown and the inorganic carbon chemistry of aquatic systems can be captured by a relatively simple model. Extrapolation of this experimentally validated model to lakes warns that rising CO_2_ levels are likely to intensify phytoplankton blooms, especially in low to moderately alkaline waters with high nutrient loads.

## Supporting Information

Text S1 **Sampling of Lake Volkerak.** Description of the lake, sampling method and analysis of the data displayed in Fig. 1.
(PDF)

Text S2 **Model description and parameter estimation.** Full description of the model used to predict the population dynamics, inorganic carbon chemistry, pH and alkalinity in the chemostat. Solubility and dissociation constants of dissolved inorganic carbon and phosphate are given in Table S2.1 in this text. System parameters and phytoplankton parameters are listed in Tables S2.2 and S2.3, respectively.
(PDF)

Text S3 **Adaptation of the model to lakes.** Detailed description of the extended model formulation to apply the model to lakes.
(PDF)

Text S4 **Dynamic changes in inorganic carbon chemistry and pH without phytoplankton.** Auxiliary experiments investigating dynamic changes in inorganic carbon chemistry and pH in six chemostats without phytoplankton. The experimental data and model fits are displayed in Fig. S4.1, and the estimated parameter values are given in Table S4.1 in this text.
(PDF)
