# Supplementary material for: Rising CO2 Levels Will Intensify Phytoplankton Blooms in Eutrophic and Hypertrophic Lakes
Source: PLoS One. 2014 Aug 13;9(8):e104325. doi: 10.1371/journal.pone.0104325 (PMC4132121; doi:10.1371/journal.pone.0104325)
Supplement: Text S1 — Sampling of Lake Volkerak. Description of the lake, sampling method and analysis of the data displayed in Fig. 1. (PDF) [file pone.0104325.s001.pdf]

Supporting Information

Text S1 Sampling of Lake Volkerak

Site description

Lake Volkerak is situated in the south-west of the Netherlands. It has a surface area of 45.7 km^2, an average depth of 5.2 m and a maximum depth of ~22 m. The area used to be a saline estuary with extensive tidal sand flats. The former Volkerak estuary was closed off from the tidal influence of the sea after completion of the Philipsdam in 1987, and turned into a large freshwater lake. From the early 1990s onwards, the lake became dominated by dense blooms of the harmful cyanobacterium *Microcystis aeruginosa*, which may comprise more than 95% of the phytoplankton community during the summer period [1,2].

Lake Volkerak is a highly eutrophic lake, with an ample supply of nitrogen and phosphorus from the surrounding agricultural land. Nitrogen concentrations never reach limiting values, while phosphorus concentrations are depleted only occasionally. The lake is very turbid throughout the year. Even during clear skies, light conditions reach limiting values within the upper 5 m of the water column. The turbidity increases with the population density of *Microcystis*, due to self-shading within the phytoplankton bloom [1,2].

Sampling and analysis

The lake was extensively sampled every two to four weeks during the period from January 2000 until December 2001. Samples were taken from the middle of the lake (station F, see Fig.1 in [2]). Temperature and pH were immediately measured from the research vessel using a Hydrolab Multi-Parameter Water Quality Instrument. Surface water samples were taken from about 0.5 m depth using a Kemmerer water sampler.

Water samples were filtered onto glass fiber filters with a pore size of 1 µm and frozen at -20 ºC. Chlorophyll *a* was extracted from freeze-dried filters using N,N-dimethylformamide for 2 h at room temperature, and the chlorophyll concentration was analyzed spectrophotometrically [3]. DIC was measured as pCO_2 in equilibrated headspace gas of acidified field samples using a Carlo Erba 1106 Elemental Analyzer according to [4]. Alkalinity and CO_2, bicarbonate and carbonate concentrations were calculated from the measured DIC concentration, pH and temperature (see Eqns (2.14) and (2.18)-(2.20) in Text S2 of the Supporting Information), taking into account the temperature dependence of the

dissociation constants (Table S2.1 in Text S2 of the Supporting Information). This showed that alkalinity of the lake was dominated by the bicarbonate concentration, and varied between 1.5 and 2.7 mEq L^{-1}. Phosphate concentrations were low compared to the DIC concentration and had a negligible effect on alkalinity. The equilibrium CO_2 concentration, $[CO_2^*]$, was calculated from Henry's law, taking into account the temperature dependence of the solubility coefficient K_0 (Table S2.1) and assuming an atmospheric CO_2 partial pressure of 370 ppm representative for the years 2000-2001.

References

1. Verspagen JMH, Snelder EOFM, Visser PM, Jöhnk KD, Ibelings BW, et al. (2005) Benthic-pelagic coupling in the population dynamics of the harmful cyanobacterium *Microcystis*. Freshw Biol 50: 854-867.
2. Verspagen JMH, Passarge J, Jöhnk KD, Visser PM, Peperzak L, Boers P, Laanbroek HJ, Huisman J (2006) Water management strategies against toxic *Microcystis* blooms in the Dutch delta. Ecol Appl 16: 313-327.
3. Porra RJ, Thompson WA, Kriedemann PE (1989) Determination of accurate extinction coefficients and simultaneous equations for assaying chlorophylls *a* and *b* extracted with four different solvents: verification of the concentration of chlorophyll standards by atomic absorption spectroscopy. Biochim Biophys Acta 975: 384-394.
4. Miyajima T, Yamada Y, Hanba YT, Yoshii K, Koitabashi T, Wada E (1995) Determining the stable isotope ratio of total dissolved carbon in lake water by GC/C/IRMS. Limnol Oceanogr 40: 994-1000.
