# Supplementary material for: Rising CO2 Levels Will Intensify Phytoplankton Blooms in Eutrophic and Hypertrophic Lakes
Source: PLoS One. 2014 Aug 13;9(8):e104325. doi: 10.1371/journal.pone.0104325 (PMC4132121; doi:10.1371/journal.pone.0104325)
Supplement: Text S2 — Model description and parameter estimation. Full description of the model used to predict the population dynamics, inorganic carbon chemistry, pH and alkalinity in the chemostat. Solubility and dissociation constants of dissolved inorganic carbon and phosphate are given in Table S2.1 in this text. System parameters and phytoplankton parameters are listed in Tables S2.2 and S2.3, respectively. (PDF) [file pone.0104325.s002.pdf]

Supporting Information

Text S2 Model description and parameter estimation

Our model builds upon a long tradition of model studies in phytoplankton ecology [1-6], extending these earlier studies by the incorporation of dynamic changes in inorganic carbon availability, alkalinity and pH induced by phytoplankton blooms. The model considers a well-mixed water column, illuminated from above, with a growing phytoplankton population that is homogeneously distributed over depth. Here, we present a detailed description of the full model as applied to the chemostat experiments. Text S3 of the Supporting Information describes how we extended the model to apply to lakes.

General outline: In this study, we assume that all nutrients are in excess. Hence, the phytoplankton growth rate does not become limited by nutrients, but is fully governed by the availability of light and inorganic carbon. The growing phytoplankton population gradually increases the turbidity of the water column, which provides an important feedback on phytoplankton bloom development by reducing the underwater light availability for photosynthesis [3,7]. Inorganic carbon is provided by dissolution of CO_2 in water and by respiratory activities of the organisms. Phytoplankton take up both CO_2 and bicarbonate for carbon assimilation [8-11], which leads to a gradual depletion of the CO_2 availability in phytoplankton blooms. Carbon and nutrient uptake by the phytoplankton population also induces dynamic changes in pH and alkalinity [12]. Changes in pH and alkalinity, in turn, affect the availability of the different inorganic carbon species [13,14], which also feeds back on phytoplankton growth.

Population dynamics: We assume that the specific growth rate of the phytoplankton depends on its cellular carbon content, adopting the structure of Droop's classic growth model [1,5,15]. The cellular carbon content is a dynamic variable, which increases by the photosynthetically-driven uptake of carbon dioxide and bicarbonate, while it decreases by respiration and by dilution of the cellular carbon content due to population growth. Let X denote the population density of the phytoplankton, and let Q denote its carbon content. Changes in phytoplankton population density and its carbon content can then be described by two coupled differential equations:

$$\frac{dX}{dt} = \left(\mu(Q) - m\right)X \tag{2.1}$$

$$\frac{dQ}{dt} = u_{CO2} + u_{HCO3} - r - \mu(Q)Q \tag{2.2}$$

where $\mu\,(Q)$ is the specific growth rate of the phytoplankton as function of its carbon content, m is the specific loss rate of the phytoplankton population (e.g., by background mortality, grazing, sedimentation), u_{CO2} and u_{HCO3} are the uptake rates of carbon dioxide and bicarbonate, respectively, and r is the respiration rate.

Carbon assimilated by phytoplankton is allocated to structural biomass and a transient carbon pool. The relative size of the transient carbon pool, T_C, is defined as:

$$T_C = \frac{Q - Q_{MIN}}{Q_{MAX} - Q_{MIN}} \tag{2.3}$$

where Q_{MIN}, is the minimum cellular carbon content that needs to be built into structural biomass in order for a cell to function, and Q_{MAX} is the maximum carbon content of a cell. The transient carbon pool can be invested into new structural biomass, which contributes to further phytoplankton growth. Hence, the specific growth rate of the phytoplankton is determined by the size of its transient carbon pool:

$$\mu(Q) = \mu_{MAX} T_C = \mu_{MAX}\left(\frac{Q - Q_{MIN}}{Q_{MAX} - Q_{MIN}}\right) \tag{2.4}$$

where μ_{MAX}, is the maximum specific growth rate. Accordingly, the specific growth rate equals zero if the transient carbon pool is exhausted (i.e., $\mu(Q_{MIN}) = 0$), and reaches its maximum if cells are satiated with carbon (i.e., $\mu(Q_{MAX}) = \mu_{MAX}$).

Photosynthesis and respiration: The light reactions of photosynthesis determine the amount of energy available for carbon fixation. We assume that the light reactions of photosynthesis are a function, $p(I)$, of the local light intensity, I:

$$p(I) = \frac{p_{MAX} I}{\left(p_{MAX}/\alpha\right) + I} \tag{2.5}$$

where p_{MAX} is the maximum photosynthetic rate of the phytoplankton, and α is the slope of the $p(I)$ curve at $I = 0$.

The light intensity, I, decreases with depth, z, according to Lambert-Beer's law:

$$I(z) = I_{IN} \exp\left(-K_{bg} z - kXz\right) \tag{2.6}$$

where I_{IN} is the incident light intensity at the top of the water column, K_{bg} is the background turbidity of the water, and k is the specific light attenuation coefficient of a phytoplankton cell. This equation includes self-shading by the phytoplankton population, because an increase in population density X will lead to a reduction in light intensity $I(z)$. We define I_{OUT} as the light intensity reaching the bottom of the water column, i.e., $I_{OUT} = I(z_{MAX})$, where z_{MAX} is the total depth of the water column.

The depth-averaged photosynthetic rate of a phytoplankton cell mixed through the water column can then be calculated from Eqns (2.5) and (2.6) as [3]:

$$P = \frac{1}{z_{\mathrm{MAX}}} \int_0^{z_{MAX}} p(I(z))\,dz$$

$$= \left(\frac{p_{MAX}}{\ln(I_{IN} / I_{OUT})} \right) \ln\left(\frac{p_{MAX} + \alpha I_{IN}}{p_{MAX} + \alpha I_{OUT}} \right) \qquad (2.7)$$

The dark reactions of photosynthesis assimilate inorganic carbon. Phytoplankton take up both CO_2 and bicarbonate for carbon assimilation. We assume that uptake rates of CO_2 and bicarbonate are increasing but saturating functions of carbon availability as in Michaelis-Menten kinetics, and are suppressed when phytoplankton cells become satiated with carbon [16]. The energy required for carbon assimilation comes from the light reactions. Uptake rates of CO_2 and bicarbonate can then be described by:

$$u_{CO2} = \left(\frac{u_{MAX,CO2}[\mathrm{CO_2}]}{H_{CO2} + [\mathrm{CO_2}]} \right) (1 - T_C)\, P \qquad (2.8)$$

$$u_{HCO3} = \left(\frac{u_{MAX,HCO3}[\mathrm{HCO_3^-}]}{H_{HCO3} + [\mathrm{HCO_3^-}]} \right) (1 - T_C)\, P \qquad (2.9)$$

where $u_{MAX,CO2}$ and $u_{MAX,HCO3}$ are the maximum uptake rates of CO_2 and bicarbonate, respectively, H_{CO2} and H_{HCO3} are the half-saturation constants, T_C is the relative size of the transient carbon pool as defined by Eqn (2.3), and P represents the depth-averaged photosynthetic rate described by Eqn (2.7). Without loss of generality, the number of model parameters can be reduced by incorporation of the maximum photosynthetic rate p_{MAX} into the maximum uptake rates of CO_2 and bicarbonate, by setting $p_{MAX} = 1$.

Carbon is lost by respiration. We assume that the respiration rate is proportional to the size of the transient carbon pool [17]:

$$r = r_{MAX}\, T_C \qquad (2.10)$$

where r_{MAX} is the maximum respiration rate when cells are fully satiated with carbon.

Level of carbon limitation: To assess to what extent phytoplankton growth is limited by carbon, we introduce a simple relative measure of the inorganic carbon availability for photosynthesis (f_C):

$$f_C = \frac{u_{MAX,CO2}\left(\frac{[\mathrm{CO_2}]}{H_{CO2}+[\mathrm{CO_2}]}\right)+u_{MAX,HCO3}\left(\frac{[\mathrm{HCO_3^-}]}{H_{HCO3}+[\mathrm{HCO_3^-}]}\right)}{u_{MAX,CO2}+u_{MAX,HCO3}} \qquad (2.11)$$

We note that $0 \le f_C \le 1$. The level of carbon limitation (L_C) can then be defined as the reduction in carbon uptake due to low carbon availability: L_C=(1-f_C)×100%. Accordingly, if CO_2 and bicarbonate are both available in saturating concentrations, f_C will be close to 1, and hence L_C will be close to 0%. Conversely, if CO_2 and bicarbonate are available only in trace amounts, L_C approaches 100%.

Dissolved inorganic carbon: On the timescales used in our model (ranging from minutes to days) the speciation of dissolved inorganic carbon is essentially in equilibrium with alkalinity and pH. Therefore, let [DIC] denote the total concentration of dissolved inorganic carbon. Changes in [DIC] can then be described by:

$$\frac{d[\mathrm{DIC}]}{dt} = D([\mathrm{DIC}]_{\mathrm{IN}} - [\mathrm{DIC}]) + \frac{g_{CO2}}{z_{MAX}} + (r - u_{CO2} - u_{HCO3})X \qquad (2.12)$$

where D is the dilution rate, g_{CO2} is the CO_2 flux rate across the air-water interface (also known as the carbon sequestration rate), and division by z_{MAX} converts the CO_2 flux per unit surface into a volumetric CO_2 change. Hence, this equation describes changes in the DIC concentration due to the influx ($[\mathrm{DIC}]_{\mathrm{IN}}$) and efflux of water containing DIC and due to gas exchange with atmospheric CO_2 (g_{CO2}). Furthermore, the DIC concentration increases through respiration (r) and decreases through uptake of CO_2 (u_{CO2}) and bicarbonate (u_{HCO3}) by phytoplankton.

We assume that the rate of CO_2 gas exchange (g_{CO2}) between air and water is proportional to the concentration gradient across the air-water interface, which can be quantified as the difference between dissolved CO_2 in equilibrium with the atmospheric pressure ($[CO_2^*]$) and the actual dissolved CO_2 concentration [18,19]:

$$g_{CO2} = v([\mathrm{CO_2^*}] - [\mathrm{CO_2}]) \qquad (2.13)$$

where v is an exchange constant. The equilibrium value $[CO_2^*]$ is calculated from Henry's law, i.e., $[CO_2^*]=K_0\ pCO_2$, where pCO_2 is the partial pressure of CO_2 in air and K_0 is the solubility constant of CO_2 gas in water. In our experiments, gas exchange will increase with

the gas flow rate (a). Hence, we assume $v = b\,a$, where b is a constant of proportionality reflecting the efficiency of gas exchange.

Alkalinity: Changes in pH depend on alkalinity, which is a measure of the acid-neutralizing capacity of water. In our experiments, alkalinity is dominated by dissolved inorganic carbon and inorganic phosphates. The alkalinity can then be described as [12,20]:

$$\mathrm{ALK} = [\mathrm{HCO_3^-}] + 2[\mathrm{CO_3^{2-}}] + [\mathrm{HPO_4^{2-}}] + 2[\mathrm{PO_4^{3-}}] + [\mathrm{OH^-}] - [\mathrm{H_3PO_4}] - [\mathrm{H^+}] \quad (2.14)$$

We note from Eqn (2.14) that changes in the concentration of dissolved CO_2 do not change alkalinity. Furthermore, uptake of bicarbonate for phytoplankton photosynthesis is accompanied by the release of a hydroxide ion or uptake of a proton to maintain charge balance, and therefore does not change alkalinity either. Hence, carbon assimilation by phytoplankton does not affect alkalinity [12]. However, nitrate, phosphate and sulfate assimilation are accompanied by proton consumption to maintain charge balance, and thus increase alkalinity [12,21]. More specifically, both nitrate and phosphate uptake increase alkalinity by 1 mole equivalent, whereas sulfate uptake increases alkalinity by 2 mole equivalents [12]. Hence, changes in alkalinity can be described as:

$$\frac{d\mathrm{ALK}}{dt} = D(\mathrm{ALK_{IN}} - \mathrm{ALK}) + (u_N + u_P + 2u_S)X \quad (2.15)$$

where $\mathrm{ALK_{IN}}$ is the alkalinity of the water influx, and u_N, u_P and u_S are the uptake rates of nitrate, phosphate and sulfate, respectively. We assume for simplicity that the uptake rates of nitrate, phosphate and sulfate are proportional to the net uptake rate of carbon:

$$u_j = c_j(u_{CO2} + u_{HCO3} - r) \qquad \text{with } j = N,P,S \quad (2.16)$$

where c_N, c_P and c_S are the cellular N:C, P:C and S:C ratio, respectively. The model keeps track of dynamic changes in the concentrations of total dissolved inorganic nitrogen ([DIN]), phosphorus ([DIP]) and sulfur ([DIS]):

$$\frac{d[\mathrm{DIN}]}{dt} = D([\mathrm{DIN}]_{\mathrm{IN}} - [\mathrm{DIN}]) - u_N X$$

$$\frac{d[\mathrm{DIP}]}{dt} = D([\mathrm{DIP}]_{\mathrm{IN}} - [\mathrm{DIP}]) - u_P X \quad (2.17)$$

$$\frac{d[\mathrm{DIS}]}{dt} = D([\mathrm{DIS}]_{\mathrm{IN}} - [\mathrm{DIS}]) - u_S X$$

where $[\mathrm{DIN}]_{\mathrm{IN}}$, $[\mathrm{DIP}]_{\mathrm{IN}}$ and $[\mathrm{DIS}]_{\mathrm{IN}}$ are the concentration of dissolved inorganic nitrogen, phosphorus and sulfur in the influx.

Algorithm to calculate dissolved CO_2, bicarbonate, carbonate and pH: The concentrations of dissolved CO_2, bicarbonate and carbonate and the pH can be calculated assuming equilibrium with [DIC], [DIP] and alkalinity [13,14]. For this purpose, we used an iterative algorithm that is solved at each time step of our model simulations. Initial estimates of the concentrations of dissolved CO_2, bicarbonate, carbonate, phosphoric acid (H_3PO_4), dihydrogen phosphate ($H_2PO_4^-$), hydrogen phosphate (HPO_4^{2-}), and phosphate (PO_4^{3-}) at a given time step in our simulations can be calculated from [DIC], [DIP] and the proton concentration ($[H^+]$) obtained from the pH at the previous time step ($pH_{t\text{-}1}$):

$$[CO_2] = \frac{[H^+]^2[DIC]}{[H^+]^2 + K_1[H^+] + K_1K_2} = \alpha_0[DIC] \qquad (2.18)$$

$$[HCO_3^-] = \frac{K_1[H^+][DIC]}{[H^+]^2 + K_1[H^+] + K_1K_2} = \alpha_1[DIC] \qquad (2.19)$$

$$[CO_3^{2-}] = \frac{K_1K_2[DIC]}{[H^+]^2 + K_1[H^+] + K_1K_2} = \alpha_2[DIC] \qquad (2.20)$$

$$[H_3PO_4] = \frac{[H^+]^3}{\alpha_P}[DIP] \qquad (2.21)$$

$$[H_2PO_4^-] = \frac{K_{P1}[H^+]^2}{\alpha_P}[DIP] \qquad (2.22)$$

$$[HPO_4^{2-}] = \frac{K_{P1}K_{P2}[H^+]}{\alpha_P}[DIP] \qquad (2.23)$$

$$[PO_4^{3-}] = \frac{K_{P1}K_{P2}K_{P3}}{\alpha_P}[DIP] \qquad (2.24)$$

Here, K_1 and K_2 are the equilibrium dissociation constants of CO_2 and bicarbonate, and K_{P1}, K_{P2} and K_{P3} are the equilibrium dissociation constants of the inorganic phosphates (Table S2.1). Furthermore, α_P is calculated as:

$$\alpha_P = [H^+]^3 + K_{P1}[H^+]^2 + K_{P1}K_{P2}[H^+] + K_{P1}K_{P2}K_{P3} \qquad (2.25)$$

A first estimate of the alkalinity can then be calculated from Eqn (2.14), using the concentrations of the inorganic carbon and phosphorus species estimated by Eqns (2.18-2.25) as input. Alternatively, alkalinity can be calculated from dynamic changes of alkalinity using Eqn (2.15). The difference, ΔALK, between the alkalinity calculated from Eqn (2.14) and the alkalinity calculated from Eqn (2.15) is used to make a new pH estimate:

$$pH_t = pH_{t\text{-}1} + \Delta pH \qquad (2.26)$$

where, ΔpH is calculated using the buffer capacity (*BC*) [13,14]:

$$\Delta\text{pH} = \frac{\Delta\text{ALK}}{BC} \tag{2.27}$$

with

$$BC = \frac{[\text{H}^+] + [\text{OH}^-] + (\alpha_1(\alpha_0 + \alpha_2) + 4\alpha_0\alpha_2)[\text{DIC}] + \alpha_{01}\alpha_{10}[\text{P}_{01}] + \alpha_{12}\alpha_{21}[\text{P}_{12}] + \alpha_{23}\alpha_{32}[\text{P}_{23}]}{{}^{10}\log(\text{e})} \tag{2.28}$$

where α_{01} = $[H^+]/([H^+] + K_{P1})$, α_{10} = $K_{P1}/([H^+] + K_{P1})$, α_{12} = $[H^+]/([H^+] + K_{P2})$, α_{21} = $K_{P2}/([H^+] + K_{P2})$, α_{23} = $[H^+]/([H^+] + K_{P3})$, α_{32} = $K_{P3}/([H^+] + K_{P3})$, $[P_{01}] = [H_3PO_4] + [H_2PO_4^-]$, $[P_{12}] = [H_2PO_4^-] + [HPO_4^{2-}]$, and $[P_{23}] = [HPO_4^{2-}] + [PO_4^{3-}]$. This new pH estimate is then used to calculate new estimates for the different species of inorganic carbon and inorganic phosphate using Eqns (2.18-2.25). This yields a new alkalinity estimate (Eqn 2.14), which gives a new pH, and so on. This iterative procedure is continued until the alkalinities calculated from Eqn (2.14) and from Eqn (2.15) have converged to the same value (and hence ΔALK and ΔpH have converged to zero). Finally, the dissolved CO_2, bicarbonate and carbonate concentrations are calculated from the resulting pH value using Eqns (2.18-2.20).

Total carbon budget: To evaluate the mass balance of carbon in the system, we can calculate the total carbon budget. The total amount of carbon in the system (C_{tot}) consists of dissolved inorganic carbon and the organic carbon contained in phytoplankton biomass. Hence, with the use of Eqns (2.1), (2.2) and (2.12), dynamic changes in the total carbon budget can be described as:

$$\frac{dC_{tot}}{dt} = \frac{d[\text{DIC}]}{dt} + \frac{d(QX)}{dt}$$

$$= D([\text{DIC}]_{\text{IN}} - [\text{DIC}]) + \frac{g_{CO2}}{z_{MAX}} - mQX \tag{2.29}$$

This equation shows that the total carbon budget changes through the influx and efflux of DIC, through the influx of CO_2 gas from the atmosphere, and through the efflux of organic carbon fixed by phytoplankton photosynthesis.

Parameter estimates: System parameters, such as the incident light intensity, mixing depth of the chemostats, composition of the mineral medium, dilution rate, and CO_2 concentration in the gas flow, were all measured prior to and/or during the experiments. They are enlisted in Table S2.2.

Some phytoplankton parameters were measured during the laboratory experiments , while others were estimated from model fits to the experimental data. We assumed that the specific loss rate of the phytoplankton was governed by the dilution rate of the chemostat (i.e., $m=D$). The number of model parameters was reduced by incorporation of the maximum photosynthetic rate p_{MAX} into the maximum uptake rates of CO_2 and bicarbonate, by setting $p_{MAX} = 1$. The cellular N:C, P:C and S:C ratios were measured experimentally. The minimum and maximum carbon contents were estimated from our measurements of the cellular carbon content. The specific light attenuation coefficient and background turbidity were estimated from Lambert-Beer's law. According to Eqn (2.6), Lambert-Beer's law can be written as $\ln(I_{IN}/I_{OUT})/z_{MAX} = K_{bg} + kX$. Hence, the specific light attenuation coefficient (k) was estimated as the slope of a linear regression of $\ln(I_{IN}/I_{OUT})/z_{MAX}$ versus the population density X, while the background turbidity (K_{bg}) was estimated as the intercept.

The remaining phytoplankton parameters were estimated by fitting the time courses predicted by the model to the time courses of the variables measured during the experiments. These measured variables included population density, cellular carbon content, light transmission I_{OUT}, dissolved CO_2, bicarbonate, carbonate and total DIC concentration, alkalinity and pH. The model fits were based on minimization of the residual sum of squares, following the same procedures as in earlier studies [6,22]. The phytoplankton parameters and their estimates are enlisted in Table S2.3.

Table S2.1. Solubility and dissociation constants of dissolved inorganic carbon and dissolved inorganic phosphates in water. The pK_a values in the table assume a pressure of 1 atm, a temperature (*θ*) of 21.5°C, and a salinity (*Sal*) of 0 g L^{-1}.

Reactions	Equilibrium constants	Description	pK_a value*	Units
$[H_2O] \leftrightarrow [H^+] + [OH^-]$	$K_W = [H^+][OH^-]$	Equilibrium constant of water	14.113[23]	-
$pCO_2 + [H_2O] \leftrightarrow [CO_2]$	$K_0 = \frac{[CO_2^*]}{pCO_2}$	Solubility of CO_2 gas in water	1.426[24]	mol L^{-1} atm^{-1}
$[CO_2] \leftrightarrow [H^+] + [HCO_3^-]$	$K_1 = \frac{[H^+][HCO_3^-]}{[CO_2]}$	Dissociation constant of CO_2	6.372[25]	-
$[HCO_3^-] \leftrightarrow [H^+] + [CO_3^{2-}]$	$K_2 = \frac{[H^+][CO_3^{2-}]}{[HCO_3^-]}$	Dissociation constant of HCO_3^-	10.362[25]	-
$[H_3PO_4] \leftrightarrow [H^+] + [H_2PO_4^-]$	$K_{P1} = \frac{[H^+][H_2PO_4^-]}{[H_3PO_4]}$	Dissociation constant of H_3PO_4	2.148	-
$[H_2PO_4^-] \leftrightarrow [H^+] + [HPO_4^{2-}]$	$K_{P2} = \frac{[H^+][HPO_4^{2-}]}{[H_2PO_4^-]}$	Dissociation constant of $H_2PO_4^-$	7.199	-
$[HPO_4^{2-}] \leftrightarrow [H^+] + [PO_4^{3-}]$	$K_{P3} = \frac{[H^+][PO_4^{3-}]}{[HPO_4^{2-}]}$	Dissociation constant of HPO_4^{2-}	12.35	-

* In all data analyses and model simulations, pK_a values were corrected for temperature and salinity according to [23-25]. The temperature and salinity data are provided in Table S2.2 in Text S2 and Table S4.1 in Text S4.

Table S2.2. System parameters used in the chemostat experiments and lake model.

Parameter	Description	*Chemostat experiments*		Lake model	Units
		Microcystis CYA140	*Microcystis* HUB5-2-4		
D	Dilution rate	0.011	0.00625	0.0003	h^{-1}
I_{IN}	Incident light intensity	50	50	400	μmol photons m^{-2} s^{-1}
K_{bg}	Background turbidity	9.5	9	1.27	m^{-1}
z_{MAX}	Depth of water column	0.05	0.05	5	m
θ	Temperature	21	24	20	°C
Sal	Salinity	L:1.23<br>H:1.36	1.23-1.36	0.1-2.6	g L^{-1}
a	Gas flow rate	25	25	–	L h^{-1}
b	Constant of proportionality for gas influx	L: 2.0×10^{-2}<br>H:1.25×10^{-2}	2.5×10^{-2}	–	m L^{-1}
pCO_2	Partial pressure of CO_2 in gas inflow	L: 200<br>H:1,200	0.5-2,800	0.1-10,000	ppm
$[DIC]_{IN}$	Concentration of DIC at influx	L:0.5<br>H:2.0	0.5-2.0	1.4×10^{-5}-10	mmol L^{-1}
ALK_{IN}	Alkalinity at influx	L:0.8<br>H:2.3	0.8-2.3	0.1-10	mEq L^{-1}
$[DIP]_{IN}$	Concentration of phosphate at influx	287	287	15	μmol L^{-1}
$[DIN]_{IN}$	Concentration of nitrate at influx	12,000	12,000	150	μmol L^{-1}
$[DIS]_{IN}$	Concentration of sulfate at influx	406	406	20	μmol L^{-1}
m	Specific mortality rate	0.011	0.00625	0.003	h^{-1}
ε	Recycling efficiency of dead phytoplankton	–	–	0.95	–
v	Gas transfer velocity of CO_2	L:0.50<br>H:0.31	0.63	0.02	m h^{-1}

L: Treatment with low pCO_2 and low bicarbonate concentration in the mineral medium

H: Treatment with high pCO_2 and high bicarbonate concentration in the mineral medium

Table S2.3. Parameter values estimated for *Microcystis* CYA140 and *Microcystis* HUB5-2-4.

Parameter	Description	*Microcystis* CYA140	*Microcystis* HUB5-2-4	Units
μ_{MAX}	Maximum specific growth rate	0.86	0.83	d^{-1}
p_{MAX}	Maximum photosynthetic rate	1	1	-
k	Specific light attenuation coefficient	L:6.9×10^{-5}<br>H:8.2×10^{-5}	6.5×10^{-5}	$m^2\ mm^{-3}$
α	Slope of the *p(I)* curve at *I* = 0	7.1×10^{-2}	5.9×10^{-2}	(μmol photons $m^{-2}\ s^{-1})^{-1}$
$u_{MAX,CO2}$	Maximum uptake rate of CO_2	8.2	4.8	μmol $mm^{-3}\ d^{-1}$
H_{CO2}	Half-saturation constant for CO_2 uptake	0.5	0.1	μmol L^{-1}
$u_{MAX,HCO3}$	Maximum uptake rate of HCO_3^-	7.3	2.6	μmol $mm^{-3}\ d^{-1}$
H_{HCO3}	Half-saturation constant for HCO_3^- uptake	75	50	μmol L^{-1}
r_{MAX}	Maximum respiration rate	1.1	1.3	μmol $mm^{-3}\ d^{-1}$
Q_{MIN}	Minimum carbon content	9	15	μmol mm^{-3}
Q_{MAX}	Maximum carbon content	17	19	μmol mm^{-3}
c_N	Cellular N:C ratio	0.18	0.153	molar ratio
c_P	Cellular P:C ratio	8.0×10^{-3}	0.0163	molar ratio
c_S	Cellular S:C ratio	7.6×10^{-3}	6.4×10^{-3}	molar ratio

L: Treatment with low pCO_2 and low bicarbonate concentration in the mineral medium

H: Treatment with high pCO_2 and high bicarbonate concentration in the mineral medium

References

1. Droop MR (1973) Some thoughts on nutrient limitation in algae. J Phycol 9: 264-272.
2. Grover JP (1991) Resource competition in a variable environment: phytoplankton growing according to the variable-internal-stores model. Am Nat 138: 811-835.
3. Huisman J, Weissing FJ (1994) Light-limited growth and competition for light in well-mixed aquatic environments: an elementary model. Ecology 75: 507-520.
4. Diehl S (2002) Phytoplankton, light, and nutrients in a gradient of mixing depths: theory. Ecology 83: 386-398.
5. Klausmeier CA, Litchman E, Levin SA (2004) Phytoplankton growth and stoichiometry under multiple nutrient limitation. Limnol Oceanogr 49: 1463-1470.
6. Passarge J, Hol S, Escher M, Huisman J (2006) Competition for nutrients and light: stable coexistence, alternative stable states, or competitive exclusion? Ecol Monogr 76: 57-72.
7. Huisman J (1999) Phytoplankton dynamics of light-limited phytoplankton microcosm experiments. Ecology 80: 202-210.
8. Giordano M, Beardall J, Raven JA (2005) CO_2 concentrating mechanisms in algae: mechanisms, environmental modulation, and evolution. Ann Rev Plant Biol 56: 99-131.
9. Martin CL, Tortell PD (2008) Bicarbonate transport and extracellular carbonic anhydrase in marine diatoms. Physiol Plant 133: 106-116.
10. Price GD, Badger MR, Woodger FJ, Long BM (2008) Advances in understanding the cyanobacterial CO_2-concentrating-mechanism (CCM): functional components, Ci transporters, diversity, genetic regulation and prospects for engineering into plants. J Exp Bot 59: 1441-1461.
11. Reinfelder JR (2011) Carbon concentrating mechanisms in eukaryotic marine phytoplankton. Ann Rev Mar Sci 3: 291-315.
12. Wolf-Gladrow DA, Zeebe RE, Klaas C, Körtzinger A, Dickson AG (2007) Total alkalinity: the explicit conservative expression and its application to biogeochemical processes. Mar Chem 106: 287-300.
13. Portielje R, Lijklema L (1995) Carbon dioxide fluxes across the air-water interface and its impact on carbon availability in aquatic systems. Limnol Oceanogr 40: 690-699.

14. Stumm W, Morgan JJ (1996) Aquatic Chemistry: Chemical Equilibria and Rates in Natural Waters. New York: Wiley-Interscience. 1022 pp.
15. Sterner RW, Elser JJ (2002) Ecological Stoichiometry: The Biology of Elements from Molecules to the Biosphere. Princeton: Princeton University Press. 584 p.
16. Morel FMM (1987) Kinetics of nutrient uptake and growth in phytoplankton. J Phycol 23: 137-150.
17. Visser PM, Passarge J, Mur LR (1997). Modelling vertical migration of the cyanobacterium *Microcystis*. Hydrobiologia 349: 99-109.
18. Siegenthaler U, Sarmiento JL (1993) Atmospheric carbon dioxide and the ocean. Nature 365: 119-125.
19. Cole JJ, Caraco NF (1998) Atmospheric exchange of carbon dioxide in a low-wind oligotrophic lake measured by the addition of SF_6. Limnol Oceanogr 43: 647-656.
20. Hofmann AF, Meysman FJR, Soetaert K, Middelburg JJ (2008) A step-by-step procedure for pH model construction in aquatic systems. Biogeosciences 5: 227–251.
21. Goldman JC, Brewer PG (1980) Effect of nitrogen source and growth rate on phytoplankton-mediated changes in alkalinity. Limnol Oceanogr 25: 352-357.
22. Huisman J, Jonker RR, Zonneveld C, Weissing FJ (1999) Competition for light between phytoplankton species: experimental tests of mechanistic theory. Ecology 80: 211-222.
23. Dickson AG, Riley JP (1979) The estimation of acid dissociation constants in seawater media from potentiometric titrations with strong base. I. The ionic product of water, K_W. Mar Chem 7: 89-99.
24. Weiss RF (1974) Carbon dioxide in water and seawater: the solubility of a non-ideal gas. Mar Chem 2: 203-215.
25. Millero FJ, Graham TB, Huang F, Bustos-Serrano H, Pierrot D (2006) Dissociation constants of carbonic acid in seawater as a function of salinity and temperature. Mar Chem 100: 80-94.
