# Supplementary material for: Rising CO2 Levels Will Intensify Phytoplankton Blooms in Eutrophic and Hypertrophic Lakes
Source: PLoS One. 2014 Aug 13;9(8):e104325. doi: 10.1371/journal.pone.0104325 (PMC4132121; doi:10.1371/journal.pone.0104325)
Supplement: Text S3 — Adaptation of the model to lakes. Detailed description of the extended model formulation to apply the model to lakes. (PDF) [file pone.0104325.s003.pdf]

Supporting Information

Text S3 Adaptation of the model to lakes

Chemostats provide ideal systems to test model predictions under highly controlled conditions at the laboratory scale. However, natural systems operate at larger temporal and spatial scales, and require different parameter settings and sometimes also other process descriptions. Here, we describe how we adapted our model to bear more resemblance to lakes. We choose parameter values representative for the summer situation in Lake Volkerak, when the lake is dominated by dense blooms of the cyanobacterium *Microcystis* [1]. The system parameters are summarized in Table S2.2 in Text S2 of the Supporting Information. The phytoplankton parameters are summarized in Table S2.3, where we used *Microcystis* HUB5-2-4 as our model species.

Description of the model

To model phytoplankton growth in lakes, we used largely the same model structure as for the chemostat (see Text S2 of the Supporting Information). In particular, we assumed highly eutrophic conditions with an ample supply of nutrients, such that phytoplankton bloom development is primarily controlled by the availability of inorganic carbon and light. However, we made the following changes:

Up-scaling: We adapted our model system from a small laboratory chemostat to a eutrophic lake. In particular, the mixing depth was increased from a chemostat of only 5 cm deep to a shallow lake of 5 m deep. The low incident light intensity of I_{IN} = 50 μmol photons m^{-2} s^{-1} in the chemostat was replaced by a much higher incident light intensity of I_{IN} = 400 μmol photons m^{-2} s^{-1} in the lake. Conversely, the very high background turbidity of the chemostat was reduced to the lower (but still fairly high) background turbidity of Lake Volkerak. The high dilution rate of the chemostat was replaced by a low turnover rate of the lake, yielding a residence time for the lake water and its constituents of ~140 days. The very high phosphate and nitrate concentrations in the mineral medium of the chemostat were reduced to a lower (but still fairly high) phosphate concentration of 15 μmol L^{-1} and a nitrate concentration of 150 μmol L^{-1} representative for hypertrophic lakes dominated by cyanobacterial blooms [1-4].

Phytoplankton losses: In the chemostats, we assumed that phytoplankton losses were dominated by the dilution rate. In lakes, however, phytoplankton are exposed to many other loss factors, such as sedimentation, grazing by zooplankton and viral lysis. We therefore included an additional loss factor to describe the changes in phytoplankton population density (X):

$$\frac{dX}{dt} = (\mu - D - m)X \qquad (3.1)$$

where μ is the specific growth rate of phytoplankton, D is the low turnover rate of the lake (i.e., the inverse of the residence time), and m is the specific mortality rate due to, e.g., sedimentation, zooplankton grazing or viral lysis.

Nutrient recycling: In chemostats, nutrients are supplied by the continuous addition of new mineral medium, while nutrients contained in phytoplankton cells washed out by dilution are lost forever. In contrast, dead phytoplankton in lakes is often mineralized, which enables the return of carbon and nutrients from dead organic matter into the DIC and nutrient pool. For simplicity, we assume that recycling of dead material is instantaneous, so that the carbon and nutrient contents of dead phytoplankton immediately enter the DIC and nutrient pools. However, we assume that the recycling efficiency is less than 100%. A fraction (1-ε) of the dead phytoplankton is permanently lost from the water column, for instance by burial in the sediment.

Hence, the dynamics of dissolved inorganic nitrogen, phosphorus and sulfur read:

$$\frac{d[\mathrm{DIN}]}{dt} = D([\mathrm{DIN}]_{\mathrm{IN}} - [\mathrm{DIN}]) - u_N X + c_N \varepsilon m Q X$$
$$\frac{d[\mathrm{DIP}]}{dt} = D([\mathrm{DIP}]_{\mathrm{IN}} - [\mathrm{DIP}]) - u_P X + c_P \varepsilon m Q X \qquad (3.3)$$
$$\frac{d[\mathrm{DIS}]}{dt} = D([\mathrm{DIS}]_{\mathrm{IN}} - [\mathrm{DIS}]) - u_S X + c_S \varepsilon m Q X$$

This equation is identical to Eqn (2.17) in Text S2 of the Supporting Information, but also includes the mineralization rate of nutrients from dead phytoplankton ($c_i \varepsilon m Q X$). Here, c_N, c_P and c_S are the molar N:C, P:C and S:C ratio, respectively, ε is the recycling efficiency, m is the specific mortality rate and Q is the carbon content of the phytoplankton. We assumed a recycling efficiency of ε = 0.95.

Alkalinity and DIC: Similarly, changes in dissolved inorganic carbon, DIC, were described by the following equation:

$$\frac{d[\mathrm{DIC}]}{dt} = D([\mathrm{DIC}]_{\mathrm{IN}} - [\mathrm{DIC}]) + \frac{g_{CO2}}{z_{MAX}} + (r - u_{CO2} - u_{HCO3} + \varepsilon mQ)X \qquad (3.2)$$

This equation is identical to Eqn (4) in the main text, but also includes the mineralization rate of organic carbon from dead phytoplankton (εmQX).

Furthermore, just as the uptake of nitrate, phosphate and sulfate by phytoplankton increases alkalinity, mineralization of these nutrients decreases alkalinity [5]. Hence, dynamic changes in alkalinity can be described as:

$$\frac{d\mathrm{ALK}}{dt} = D(\mathrm{ALK}_{\mathrm{IN}} - \mathrm{ALK}) + (u_N + u_P + 2u_S)X - (c_N + c_P + 2c_S)\varepsilon mQX \qquad (3.4)$$

This equation is identical to Eqn (7) in the main text, but also includes effects of nutrient mineralization on alkalinity.

In our model simulations, we compare lakes with different alkalinities (Fig. 5), where alkalinity of the inflowing water (ALK_{IN}) was treated as system parameter. The DIC concentration of the inflowing water (DIC_{IN}) was calculated from ALK_{IN} and $[DIP]_{IN}$ assuming equilibrium with the atmospheric pCO_2. Furthermore, we assumed that salinity consisted of a baseline salinity of 0.1 g L^{-1} plus additional salinity due to the alkalinity of the inflowing water (assuming that alkalinity was determined by sodium bicarbonate).

Gas transfer across the air-water interface: Similar to the chemostat experiments, we assume that CO_2 gas exchange is proportional to the difference between the expected concentration of dissolved CO_2 in equilibrium with the atmosphere (calculated from Henry's law) and the actual concentration of dissolved CO_2 [6,7]:

$$g_{\mathrm{CO2}} = v(\mathrm{K}_0\, p\mathrm{CO}_2 - [\mathrm{CO}_2]) \qquad (3.5)$$

where v is the gas transfer velocity (also known as piston velocity) across the air-water interface, K_0 is the solubility constant of CO_2 gas in water, and pCO_2 is the partial pressure of CO_2 in the atmosphere. The gas transfer velocity depends on several parameters, especially wind speed. A typical value for the gas transfer velocity of lakes is v = 0.02 m h^{-1} [8,9].

References

1. Verspagen JMH, Passarge J, Jöhnk KD, Visser PM, Peperzak L, et al. (2006) Water management strategies against toxic *Microcystis* blooms in the Dutch delta. Ecol Appl 16: 313-327.
2. Jöhnk KD, Huisman J, Sharples J, Sommeijer B, Visser PM, et al. (2008) Summer heatwaves promote blooms of harmful cyanobacteria. Glob Chang Biol 14: 495-512.

3. Xu H, Paerl HW, Qin B, Zhu G, Gao G (2010) Nitrogen and phosphorus inputs control phytoplankton growth in eutrophic Lake Taihu, China. Limnol Oceanogr 55: 420-432.
4. Balmer MB, Downing JA (2011) Carbon dioxide concentrations in eutrophic lakes: undersaturation implies atmospheric uptake. Inland Waters 1: 125-132.
5. Wolf-Gladrow DA, Zeebe RE, Klaas C, Körtzinger A, Dickson AG (2007) Total alkalinity: The explicit conservative expression and its application to biogeochemical processes. Mar Chem 106: 287-300.
6. Siegenthaler U, Sarmiento JL (1993) Atmospheric carbon dioxide and the ocean. Nature 365: 119-125.
7. Cole JJ, Caraco NF (1998) Atmospheric exchange of carbon dioxide in a low-wind oligotrophic lake measured by the addition of SF_6. Limnol Oceanogr 43: 647-656.
8. Crucius J., Wanninkhof R (2003) Gas transfer velocities measured at low wind speed over a lake. Limnol Oceanogr 48: 1010-1017.
9. Guérin F, Abril G, Serça D, Delon C, Richard S, et al. (2007) Gas transfer velocities of CO_2 and CH_4 in a tropical reservoir and its river downstream. J Mar Syst 66: 161-172.
