# Supplementary material for: Rising CO2 Levels Will Intensify Phytoplankton Blooms in Eutrophic and Hypertrophic Lakes
Source: PLoS One. 2014 Aug 13;9(8):e104325. doi: 10.1371/journal.pone.0104325 (PMC4132121; doi:10.1371/journal.pone.0104325)
Supplement: Text S4 — Dynamic changes in inorganic carbon chemistry and pH without phytoplankton. Auxiliary experiments investigating dynamic changes in inorganic carbon chemistry and pH in six chemostats without phytoplankton. The experimental data and model fits are displayed in Fig. S4.1, and the estimated parameter values are given in Table S4.1 in this text. (PDF) [file pone.0104325.s004.pdf]

Supporting Information

Text S4 Dynamic changes in inorganic carbon chemistry and pH without phytoplankton

Description of experiments

Before we ran our chemostat experiments with the cyanobacterium *Microcystis,* we first studied dynamic changes in inorganic carbon chemistry and pH in six chemostats without phytoplankton. In our experiments, alkalinity is dominated by dissolved inorganic carbon and inorganic phosphates. The alkalinity can then be described as [1]:

$$\mathrm{ALK} = \left[\mathrm{HCO_3^-}\right] + 2\left[\mathrm{CO_3^{2-}}\right] + \left[\mathrm{HPO_4^{2-}}\right] + 2\left[\mathrm{PO_4^{3-}}\right] + \left[\mathrm{OH^-}\right] - \left[\mathrm{H_3PO_4}\right] - \left[\mathrm{H^+}\right] \qquad (4.1)$$

To obtain different alkalinities, we added 5, 25 or 75 mmol L^{-1} of inorganic phosphate buffer to the mineral medium (see Table S4.1 below). Prior to the experiments, the chemostats were flushed for > 4 h with pure N_2 gas, to deplete the dissolved inorganic carbon (DIC) concentration in the mineral medium. Subsequently, we added different concentrations of sodium bicarbonate, to obtain initial DIC concentrations ranging from 0.4 to 18.5 mmol L^{-1} (Table S4.1). Temperature was maintained at 21 ± 0.6ºC. During the experiments, chemostats were aerated with gas containing 50, 400, 1600 or 2800 ppm CO_2 (Table S4.1).

We set the dilution rate to zero. In this case, according to Eqn (7) in the main text, there are no changes in alkalinity. Furthermore, according to Eqn (4) and Eqn (5) in the main text, the changes in DIC concentration simplify to:

$$\frac{d[\mathrm{DIC}]}{dt} = \frac{v}{z_{MAX}}\left(K_0 \ p\mathrm{CO_2} - [\mathrm{CO_2}]\right) \qquad (4.2)$$

where v is the gas transfer velocity, K_0 is the solubility constant of CO_2 gas in water, and pCO_2 is the partial pressure of CO_2 in the gas flow. That is, changes in the total DIC concentration are fully governed by dissolution and evasion of CO_2 gas. In our experiments, gas exchange will increase with the gas flow rate (*a*). Hence, we assume $v = b\, a$, where *b* is a constant of proportionality reflecting the efficiency of gas exchange. Dynamic changes in dissolved CO_2, bicarbonate, carbonate and pH were calculated with the algorithm in Text S2.

The resulting changes in pH and DIC were monitored by taking samples every 15-30 min for a total of 4 h. DIC concentrations were determined by sampling 15 mL of culture suspension, which was immediately filtered over 0.45 μm membrane filters (Whatman, Maidstone, UK). DIC was analyzed by a Model 700 TOC Analyzer (OI Corporation, College Station, TX, USA). Temperature and pH were measured with a pH meter (SCHOTT AG, Mainz, Germany).

Parameterization of the model

System parameters, such as the gas flow rate, pCO_2 concentration in the gas flow, temperature, and the alkalinity and dissolved inorganic phosphorus (DIP) in the mineral medium were all experimentally controlled (Table S4.1). The only 'free' parameter was the constant of proportionality for the gas influx (*b*), which was estimated by fitting the time courses predicted by the model to measured time courses of DIC and pH. Model fits were based on minimization of the residual sum of squares, following the same procedures as earlier studies [2,3].

Table S4.1 System parameters used in the chemostat experiments without phytoplankton[a].

Chemostat	pCO_2 (ppm)	ALK (mEq L^{-1})	[DIP] (mmol L^{-1})	$[DIC]_{t=0}$ (mmol L^{-1})	$pH_{t=0}$	*b* (m L^{-1})	*Salinity* (‰)
a	50	75.6	75.0	0.4	9.2	1.4×10^{-1}	14.2
b	400	75.8	75.0	0.5	9.3	8.6×10^{-2}	14.2
c	400	25.6	25.0	1.1	8.5	1.4×10^{-1}	5.5
d	1600	27.0	25.0	2.0	8.8	1.4×10^{-1}	5.5
e	1600	29.5	5.0	18.5	9.5	2.1×10^{-1}	2.0
f	2800	12.5	5.0	6.0	9.3	1.4×10^{-1}	2.0

[a] Other parameters: dilution rate $D = 0$, temperature $\theta = 21$°C, gas flow rate $a = 25$ L h^{-1}

Results

Dissolution of CO_2 increased the DIC concentration and decreased pH (Fig. S4.1), until the dissolved CO_2 concentration approached equilibrium with the gas pressure (i.e., $[CO_2] = K_0\ pCO_2$). As expected, low pCO_2 and high alkalinity caused only a minor increase in DIC and minor decrease in pH (Fig. S4.1A). At intermediate pCO_2, the time course of DIC and pH depended on alkalinity and the initial DIC concentration (Fig. S4.1B-E). The strongest reduction in pH was observed in the chemostat with the highest pCO_2 in the gas flow and lowest alkalinity in the mineral medium (Fig. S4.1F). The model captured the dynamics of DIC and pH quite well.

We observed some precipitates in the experiments of Fig. S4.1E and S4.1F, most likely due to precipitation of phosphate and carbonate with divalent cations (e.g., Ca^{2+}, Mg^{2+}) in the mineral medium. Such precipitation would decrease alkalinity and hence lower the pH in the experiments [1,4], which might explain the difference between the modeled and measured pH (Fig. S4.1E,F). In our subsequent experiments with *Microcystis* described in the main text, we used much lower phosphate and carbonate concentrations and did not observe precipitates.

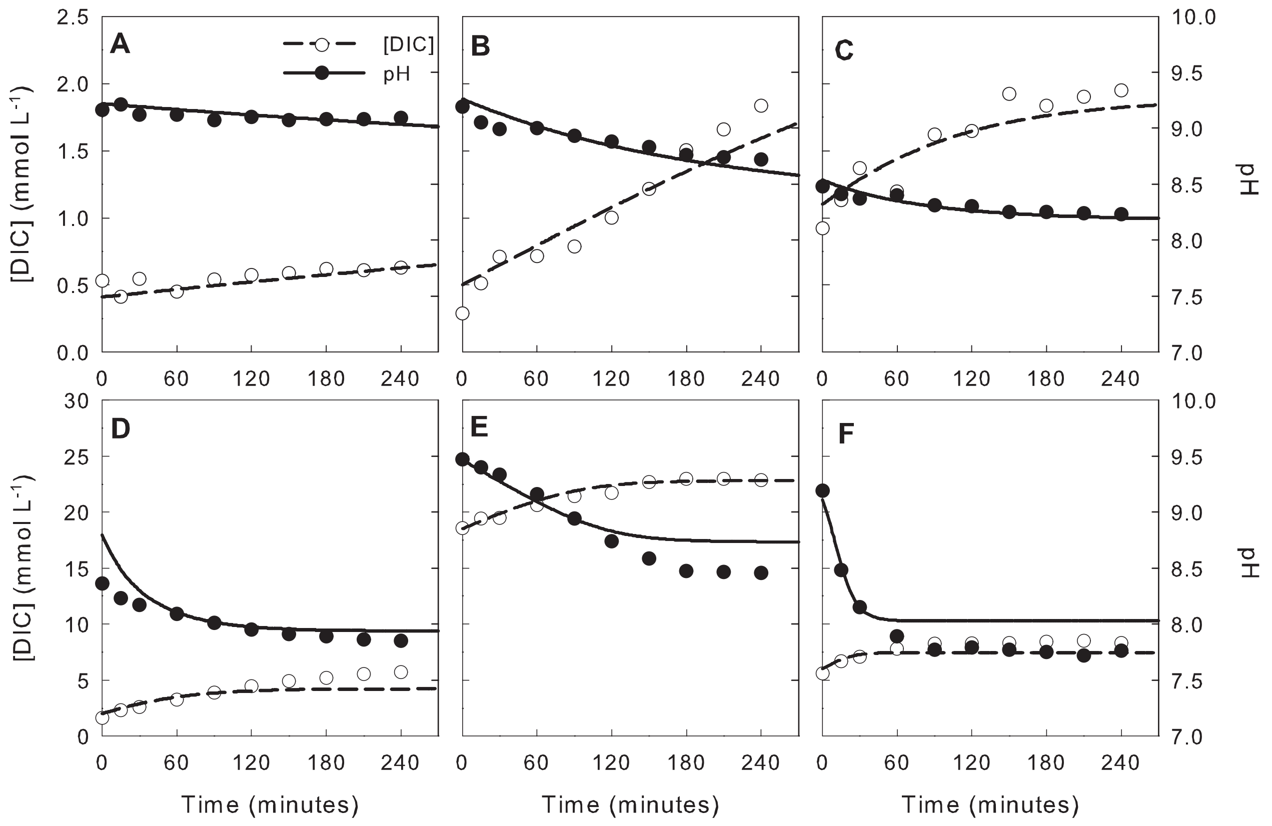

Fig. S4.1 Dynamic changes in pH and DIC concentration in chemostats without phytoplankton. The six chemostats were exposed to different pCO_2 concentrations in the gas flow and different alkalinities in the mineral medium. (A) pCO_2 = 50 ppm, ALK = 75.6 mEq L^{-1}; (B) pCO_2 = 400 ppm, ALK = 75.8 mEq L^{-1}; (C) pCO_2 = 400 ppm, ALK = 25.6 mEq L^{-1}; (D) pCO_2 = 1600 ppm, ALK = 27.0 mEq L^{-1}; (E) pCO_2 = 1600 ppm, ALK = 29.5 mEq L^{-1}; (F) pCO_2 = 2800 ppm, ALK = 12.5 mEq L^{-1}. Symbols represent measurements, lines show the model predictions based on the parameter estimates provided in Table S4.1. Note that the DIC concentration in the 3 top panels is plotted on another scale than in the 3 bottom panels.

References

1. Wolf-Gladrow DA, Zeebe RE, Klaas C, Körtzinger A, Dickson AG (2007) Total alkalinity: the explicit conservative expression and its application to biogeochemical processes. Mar Chem 106: 287-300.
2. Huisman J, Jonker RR, Zonneveld C, Weissing FJ (1999) Competition for light between phytoplankton species: experimental tests of mechanistic theory. Ecology 80: 211-222.
3. Passarge J, Hol S, Escher M, Huisman J (2006) Competition for nutrients and light: stable coexistence, alternative stable states, or competitive exclusion? Ecol Monogr 76: 57-72.
4. Stumm W, Morgan JJ (1996) Aquatic Chemistry: Chemical Equilibria and Rates in Natural Waters. Wiley-Interscience, New York, 1022 p.
